# Light‐Triggered CRISPR/Cas12a for Genomic Editing and Tumor Regression

**DOI:** 10.1002/anie.202502892

**Published:** 2025-05-19

**Authors:** Hong Liu, Jiantong Dong, Renzhi Wu, Jun Dai, Xiaoding Lou, Fan Xia, Itamar Willner, Fujian Huang

**Affiliations:** ^1^ State Key Laboratory of Geomicrobiology and Environmental Changes Faculty of Materials Science and Chemistry China University of Geosciences Wuhan 430074 China; ^2^ The Institute of Chemistry, The Center for Nanoscience and Nanotechnology The Hebrew University of Jerusalem Jerusalem 91904 Israel; ^3^ Department of Obstetrics and Gynecology, Tongji Hospital, Tongji Medical College Huazhong University of Science and Technology Wuhan 430034 China

**Keywords:** Apoptosis, Gene repairing, Machinery, Photoresponsive nucleic acid, Spatiotemporal

## Abstract

A photo‐triggered CRISPR/Cas12a machinery for in vitro and in vivo gene editing is introduced. The system consists of a caged, inactive *ortho*‐nitrobenzyl phosphate ester photo‐responsive crRNA, which, upon light‐induced deprotection, yields the active CRISPR/Cas12a gene editing machinery (LAC12aGE). The LAC12aGE system induces specific thymidine‐rich (TTTN) protospacer‐adjacent motif (PAM)‐guided double‐stranded breaks in genomic DNA, which upon non‐homologous end‐joining lead to gene repair. The LAC12aGE machinery is applied for gene editing of an exogenous dual fluorescent reporter gene in living cells, as well as the endogenous gene encoding DNA methyltransferase 1. In addition, the LAC12aGE is applied for in vitro gene editing and disruption of the hepatocyte growth factor (*HGF*) gene in HepG2 cells, where knockout of the *HGF* gene results in inhibited cell proliferation and migration, as well as enhanced apoptosis. Moreover, the in vivo knockout and disruption of the *HGF* gene in HepG2 tumors by the LAC12aGE machinery is demonstrated. The cyclic temporal development of the LAC12aGE system in tumors shows effective inhibition of tumor growth and enhanced apoptosis/necrosis of tumor tissues compared to control systems.

## Graphical abstract

A photo‐triggered CRISPR/Cas12a machinery for gene editing is introduced. The machinery is applied for endogenous DNA methyltransferase 1 (*DNMT1*) gene editing and for in vitro disruption of the hepatocyte growth factor (*HGF*) gene. The machinery is used for in vivo knockout and disruption of the *HGF* gene in HepG2 tumors, resulting in effective apoptosis and necrosis of the tumor tissues.

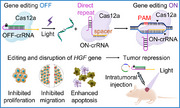

## Introduction

Clustered regularly interspaced short palindromic repeats (CRISPR)‐associated proteins (Cas) provide versatile tools for genome manipulation.^[^ ^1^ ^]^ Cas9 and Cas12a are RNA‐guided class 2 nucleases that can be programmed with a guide RNA (gRNA) to bind and cleave complementary target DNA strands.^[^ ^2^, ^3^ ^]^ As the gRNA sequence can be predesigned, the resulting CRISPR/Cas conjugates are versatile catalytic tools for genome editing and gene regulation.^[^ ^4^ ^]^ Indeed, the CRISPR systems were broadly applied for gene editing, transcriptional regulation, epigenetic modification, therapeutics, diagnostics, and more.^[^ ^5^, ^6^, ^7^, ^8^, ^9^, ^10^ ^]^ The Cas12a system reveals several advantages over the Cas9 system: (i) While the Cas9 system is limited to guanosine‐dependent protospacer adjacent motif (PAM) regions, the Cas12a system recognizes thymidine‐rich PAM domains, thereby extending the genomic editing regions.^[^ ^11^ ^]^ (ii) The Cas12a and accompanying gRNA exhibit lower molecular weights compared to the Cas9 system,^[^ ^3^ ^]^ thus enhancing transfection efficiencies and multiple gene editing capacities.^[^ ^12^, ^13^, ^14^ ^]^ (iii) The Cas12a system stimulates PAM‐distal region cutting, generating 5′‐overhang sticky ends rather than blunt‐ended breaks formed by the Cas9 system, thereby resulting in predictable repair programs by the Cas12a as compared to variable repair products by the Cas9 system.^[^ ^15^, ^16^ ^]^

An important challenge in the application of CRISPR/Cas machineries for gene editing and gene regulation, however, involves the precise spatiotemporal activation of the systems in biological environments. Indeed, different low‐molecular‐weight agents, physiological triggers, or light have been implemented to activate the CRISPR/Cas machineries.^[^ ^17^ ^]^ Light is a particularly attractive signal to control the CRISPR/Cas machineries due to the precise localization and tunable dose of the triggering light‐source, and more importantly, because it is a “clean” trigger that does not require added components or the generation of “waste” products.^[^ ^18^ ^]^ Gating of oligonucleotide structures by photo‐responsive units and photochemical uncaging of active oligonucleotide structure have an important impact on the development of dynamic DNA nanotechnology.^[^ ^19^, ^20^ ^]^ Indeed, photochemical control over the CRISPR/Cas system was accomplished by either caging of the gRNA with photo‐responsive protecting groups,^[^ ^21^, ^22^, ^23^, ^24^, ^25^, ^26^, ^27^, ^28^ ^]^ engineering the Cas protein fragments with photoinducible dimerization domains,^[^ ^29^, ^30^, ^31^ ^]^ or controlling Cas protein expression by photoactive transcription activator.^[^ ^32^, ^33^ ^]^ Different strategies have been developed to cage the gRNA and prohibit its association with the target duplex DNA and the cleaving activity of CRISPR complex.^[^ ^21^, ^22^, ^23^, ^24^, ^25^, ^26^, ^27^, ^28^ ^]^ These strategies include the chemical modification of nucleobases (uridine or guanosine) with photocleavable protecting groups (6‐nitropiperonyloxymethylene, NPOM),^[^ ^21^, ^22^, ^23^ ^]^ the terminus conjugation of bulky hydrophobic molecules (vitamin E) to the gRNA via photocleavable bridging linkers,^[^ ^24^ ^]^ crosslinking of the gRNA constituents by photocleavable units ([7‐(diethylamino)coumarin‐4‐yl]‐methyl (DEACM) linker, nitrobenzyl‐based linker, or (1′‐propargyl‐*o*‐nitrobenzyloxy)methoxyl (NPBOM) unit),^[^ ^25^, ^26^ ^]^ and blocking the gRNA with complementary oligonucleotides modified with photocleavable groups.^[^ ^27^, ^28^ ^]^ Alternatively, split Cas protein fragments were fused with photoinducible dimerization constituents (pMag and nMag) that upon photo‐crosslinking lead to an integrated, fused, active Cas protein.^[^ ^29^, ^30^, ^31^ ^]^ Moreover, the far‐red light activation of bacterial photoreceptor BphS, which converts guanosine triphosphate (GTP) into c‐di‐GMP and induces the dimerization of the transcription activator p65‐VP64‐BldD, binding to its promoter and initiating the expression of Cas proteins for gene editing, was demonstrated.^[^ ^32^, ^33^ ^]^ While the caging of the gRNA units involves fine‐tuning regarding the number of gating units, the fusion of Cas subunits requires protein engineering. Diverse applications of the photochemically triggered CRISPR/Cas9 and CRISPR/Cas12a machineries were reported, including in vitro gene editing and sensing.^[^ ^34^, ^35^, ^36^, ^37^, ^38^ ^]^ Nevertheless, the application of photo‐triggered CRISPR/Cas machineries in mammals is underdeveloped and has used either upconversion nanoparticle carriers,^[^ ^39^ ^]^ photo‐triggered fused Cas protein subunits, or photoinducible expression of Cas protein.^[^ ^31^, ^32^, ^33^ ^]^ Moreover, the in vivo photochemically‐triggered uncaging of crRNA to activate CRISPR/Cas12a for cancer therapy is unprecedented.

Here, we wish to report on the development of a light‐activated CRISPR/Cas12a gene‐editing (LAC12aGE) machinery for in vitro and in vivo gene editing. Specifically, exogenous gene expression and endogenous gene editing are demonstrated, and the LAC12aGE machinery is applied for the knockout of hepatocyte growth factor (*HGF*) gene in cells. The later process is used for spatiotemporal light‐induced inhibition of cell proliferation and migration, and the concomitant induction of apoptosis in cancer cells. The LAC12aGE machinery is further applied to suppress tumor growth in mice. It is important to note that light‐triggered activation of the CRISPR/Cas12a system for in vivo gene editing is less developed as compared to the CRISPR/Cas9 system, despite the intrinsic advantages of Cas12a, including its extended editing capacity. Moreover, the novelty of this study is highlighted by two key advancements within the LAC12aGE platform: (i) a simpler and technologically easier approach to implement by preserving an unmodified spacer region, enabling seamless adaptation to various targets without requiring additional optimization; (ii) the first demonstration of in vivo *HGF* gene knockout and the subsequent apoptosis in cancer cells for tumor therapy. To date, light‐activated CRISPR/Cas12a systems have been primarily restricted to gene activation (e.g., luciferase expression),^[^ ^31^, ^33^ ^]^ without progressing toward in vivo therapeutic applications. Our study addresses this limitation, advancing the potential of light‐regulated CRISPR/Cas12a for precise and effective therapeutic gene editing.

## Results and Discussion

### Principle of the LAC12aGE Machinery

**Figure 1:**
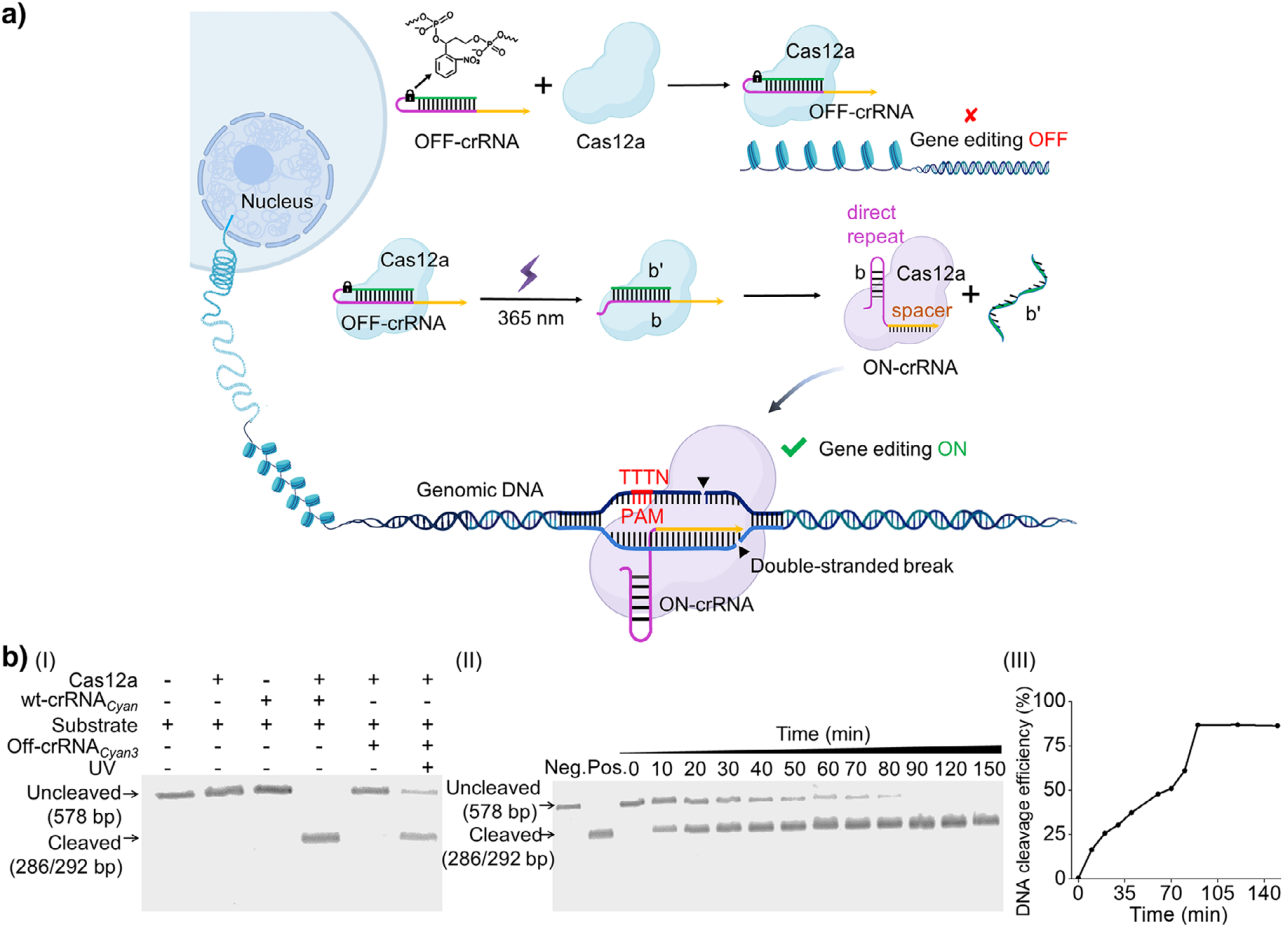
The light‐activated CRISPR/Cas12a gene editing (LAC12aGE) system. a) Schematic assembly of the Cas12a/OFF‐crRNA complex involving the light‐induced cleavage of the caged OFF‐crRNA into the pre‐crRNA duplex b/b’, yielding the Cas12a/ON‐crRNA complex. The interaction of the Cas12a/ON‐crRNA complex with the PAM (TTTN) gene editing region leads to double‐stranded breaks in the genome, facilitating non‐homologous end joining‐based gene repair. b) Electrophoretic characterization of the LAC12aGE‐induced in vitro editing of the Cyan fluorescent protein gene fragment. Panel I, Electrophoresis‐separated products of the intact LAC12aGE machinery‐edited Cyan fluorescent protein gene fragment, compared to a set of control systems. Panel II, Time‐dependent electrophoretic cleaved products generated upon subjecting the Cyan fluorescent protein gene to the LAC12aGE machinery. Neg., negative control using Cas12a in the absence of crRNA*_Cyan_*. Pos., positive control using Cas12a and wt‐crRNA*_Cyan_*. Panel III, Time‐dependent cleavage efficiency of the *Cyan* gene by the LAC12aGE machinery. The DNA cleavage efficiency (%) was calculated as 100% × [1‐(1‐fraction cleaved)^1/2^], where fraction cleaved is equal to the concentration of the cleaved DNA divided by the total concentration of DNA.

The principle of assembly of the LAC12aGE machinery is displayed in Figure 1a. An *ortho*‐nitrobenzyl phosphate ester‐protected hairpin that includes the CRISPR RNA (crRNA) in a caged configuration is photo‐deprotected (*λ* = 365 nm) in the presence of the Cas12a protein, forming the pre‐crRNA duplex b/b’. The Cas12a dissection of pre‐crRNA yields the ON‐crRNA with intact stem‐loop structure of the direct repeat region, resulting in the activated LAC12aGE machinery. The LAC12aGE machinery induces specific thymidine‐rich (TTTN) protospacer‐adjacent motif (PAM)‐dependent double‐stranded breaks (DSBs) in genomic DNA, generating 5′ overhang sticky‐end fragments curve that facilitate non‐homologous end joining (NHEJ)‐based gene repair.^[^ ^3^, ^40^, ^41^ ^]^ (For gel electrophoresis validation of the formation of Cas12a/OFF‐crRNA complex and its phototriggered activation into the active Cas12a/ON‐crRNA complex, see Figure S1.) This machinery is applied for the in vitro editing of the Cyan fluorescent protein gene fragment (Table S1). This gene includes a large number of PAM sites (5′‐TTTN‐3′), and thus could be recognized by the CRISPR/Cas12a. The optimizations of the photo‐responsive caging site within the OFF‐crRNA sequence (Table S2) are summarized in Figures S2 and S3 and the accompanying discussion. The inactive OFF‐crRNA*_Cyan3_* was selected as the precursor‐yielding crRNA for the light‐stimulated activation of the LAC12aGE machinery. The light‐stimulated activation of the LAC12aGE machinery (*λ* = 365 nm, 25 mW cm^−2^) for 5 min led to the cleavage of the 578 bp *Cyan* gene into two fragments composed of 286 and 292 bp. Notably, irradiation of the LAC12aGE machinery for a duration of 5 min resulted in complete deprotection and cleavage, as demonstrated in Figure S4. (For gel electrophoresis comparison of Cas12a/OFF‐crRNA with the always‐blocked OFF‐crRNA lacking a photo‐responsive site, see Figure S5. For optimization of the Cas12a/crRNA concentration for in vitro gene editing, see Figure S6.) Figure 1b depicts the gel electrophoretic results corresponding to the temporal cleavage of the *Cyan* gene by the LAC12aGE machinery and control systems, panel I. The negative control of non‐illuminated LAC12aGE system does not yield any fragmented products, whereas the illuminated LAC12aGE machinery reveals complete cleavage of the gene after operating the machinery for a time interval of 90 min. Moreover, the results demonstrate a temporal increase in cleavage products and a concomitant depletion of the parent gene, panel II. The contents of the cleaved gene products and the parent gene were evaluated by quantitative analysis of the respective electrophoretic bands, and the results corresponding to the temporal gene cleavage efficiencies are presented in panel III.^[^ ^24^ ^]^ An in vitro DNA cleavage efficiency of ca. 86% after 90 min of operation is demonstrated.

### LAC12aGE‐Mediated Gene Editing Toward Exogenous Gene in Living Cells

**Figure 2:**
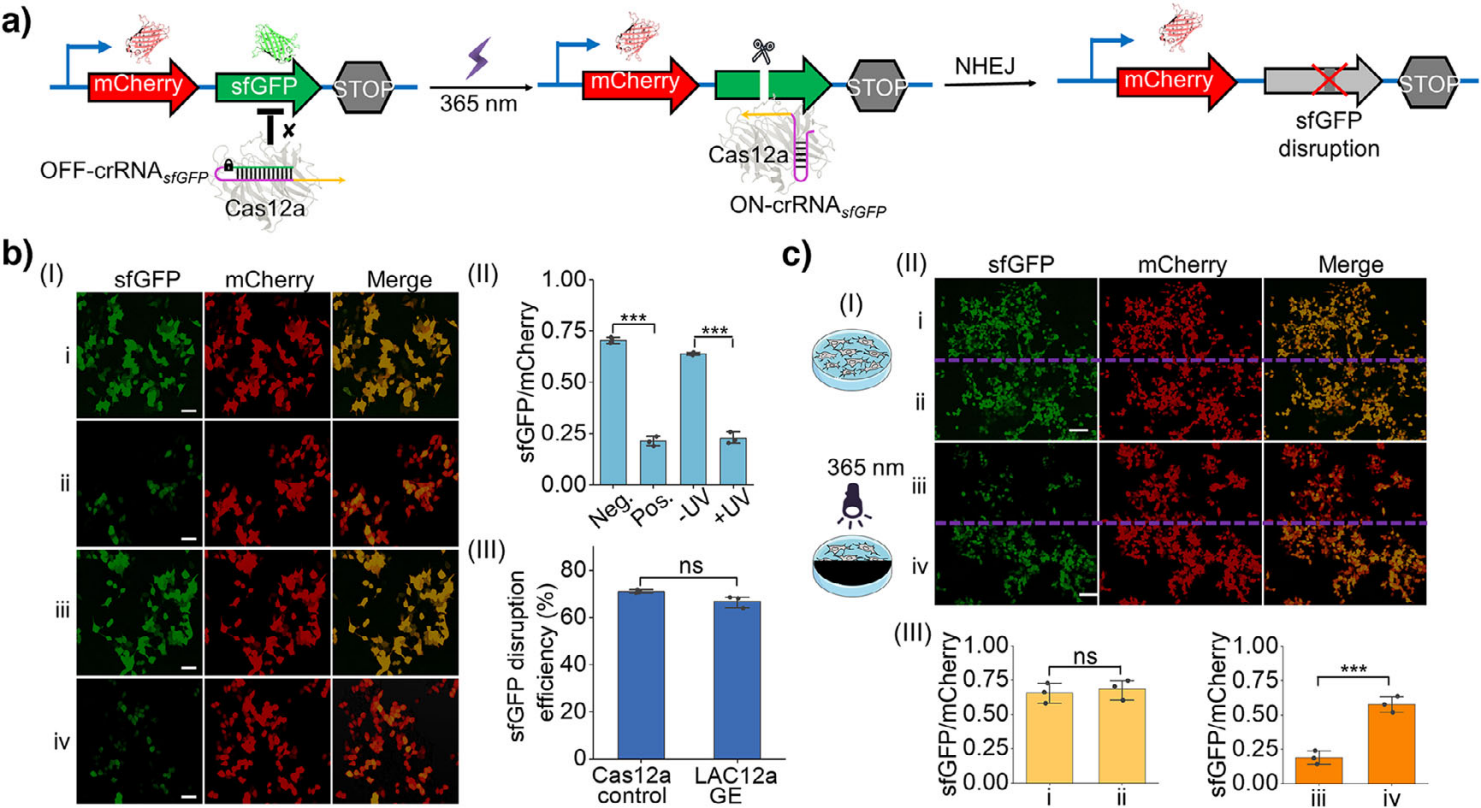
LAC12aGE‐mediated gene editing toward an exogeneous dual fluorescent reporter gene in living HEK293T cells. a) Schematic of LAC12aGE‐mediated gene editing of an exogenous dual fluorescent reporter gene. Light‐activated, Cas12a‐mediated gene editing proceeds at a predesigned locus, followed by the cellular NHEJ DNA repair pathway. The *sfGFP* expression is disrupted through NHEJ‐mediated indel mutations. b) *sfGFP* gene knockout in HEK293T cells transfected with the LAC12aGE machinery and the fluorescent dual reporter plasmid. Panel I: Confocal microscopy images corresponding to the fluorescence of *sfGFP* and mCherry in HEK293T cells. Entry i, Negative control (Neg.) transfected with Cas12a plasmid in the absence of crRNA*_sfGFP_*. Entry ii, Positive control (Pos.) transfected with Cas12a plasmid and wt‐crRNA*_sfGFP_*. Entry iii, Cells transfected with Cas12a plasmid/OFF‐crRNA*_sfGFP_* in the absence of UV light (−UV). Entry iv, Cells transfected with Cas12a plasmid/OFF‐crRNA*_sfGFP_* in the presence of UV light (+UV). Scale bar, 50 µm. Panel II: The relative ratios of *sfGFP* to mCherry derived from the integrated fluorescence intensities of *sfGFP* and mCherry displayed in panel I. Panel III: Disruption efficiencies of *sfGFP* by the Cas12a positive control and the LAC12aGE machinery, where the *sfGFP* disruption efficiency (%) by the Cas12a is defined as (Flu_Neg._–Flu_Pos._)/Flu_Neg_. × 100%, and the disruption efficiency (%) by the LAC12aGE is defined as (Flu_−UV_ – Flu_+UV_)/Flu_−UV_ × 100%. c) Panel I: Spatial control of LAC12aGE machinery using a photomasked pattern. Panel II: Confocal microscopy images corresponding to fluorescence of *sfGFP* and mCherry in HEK293T cells transfected with Cas12a/OFF‐crRNA*_sfGFP_*. Regions i and ii, Cells in the absence of a photomasked pattern and without UV light activation. Region iii, Cells in the absence of a photomasked pattern exposed to UV light illumination. Region iv, Cells in the presence of a photomasked pattern exposed to UV light illumination. Scale bars, 100 µm. Panel III: The relative ratios of *sfGFP* to mCherry derived from the integrated fluorescence intensities of *sfGFP* and mCherry displayed in panel II. ns, no significant difference; ***, *P* < 0.001. Data are presented as mean ± SD (*n *= 3).

In the next step, the LAC12aGE‐mediated gene editing capacities toward an exogenous dual fluorescent reporter gene in living cells were examined, Figure 2. Dual fluorescent reporter plasmids are commonly used as experimental tools to characterize the functions and properties of target genes or proteins.^[^ ^42^ ^]^ We visualized that the LAC12aGE exogenous gene editing capacities could be demonstrated by applying the *sfGFP* gene in the *sfGFP*/mCherry dual fluorescent reporter plasmid as the target site and the OFF‐crRNA*_sfGFP_* as a photo‐responsive caged constituent for the LAC12aGE machinery, Figure 2a. The dual fluorescent reporter plasmid is subjected to the LAC12aGE machinery in the presence of the OFF‐crRNA*_sfGFP_*, which, upon photochemical uncaging of the OFF‐crRNA*_sfGFP_*, activates the cleavage of the *sfGFP* gene, leading to the disruption of gene functions. The subsequent cellular NHEJ–DNA repair pathway leads to non‐homologous joining‐mediated indel mutations^[^ ^41^ ^]^ within the coding *sfGFP* expression region, resulting in the disruption of *sfGFP* expression. Accordingly, the dual fluorescent reporter plasmid, the Cas12a plasmid, and the OFF‐crRNA*_sfGFP_* were transfected into HEK293T cells to probe the gene editing capacities of the LAC12aGE machinery. (The in vitro capacity of the OFF‐crRNA*_sfGFP_* to cleave the 1949 bp *sfGFP* gene into two fragments of 1058 and 891 bp was demonstrated in Figure S7). Figure 2b, panel I depicts the fluorescent confocal microscopy image of the LAC12aGE‐mediated *sfGFP* gene disruption in HEK293T cells and control systems after light activation for 5 min and operation of the LAC12aGE for a time‐interval of 72 h. Entry i depicts the fluorescent features of the negative control, consisting of the cells treated with Cas12a plasmid in the absence of the active crRNA*_sfGFP_*, showing the appearance of the *sfGFP* and mCherry fluorescence, indicating no disruption of *sfGFP* gene. Entry ii depicts the positive control of cells transfected with the Cas12a plasmid and the pre‐synthesized active wt‐crRNA*_sfGFP_*. The fluorescence of *sfGFP* is depleted, indicating the Cas12a/crRNA*_sfGFP_*‐stimulated disruption of the *sfGFP* gene, while the mCherry fluorescence is observed, implying the activity of the mCherry gene expression. Entry iii and iv present the fluorescent images of cells loaded with the Cas12a plasmid and the caged OFF‐crRNA*_sfGFP_*, in the absence of exposure to light and after exposure to light (*λ *= 365 nm, 25 mW cm^−2^) for 5 min for activating the crRNA*_sfGFP_*, respectively. The cells not exposed to light reveal the fluorescence of *sfGFP* and mCherry, consistent with the non‐disrupted structure of the two genes. In contrast, the illuminated cells reveal the depletion of the *sfGFP* fluorescence and the clear fluorescence of mCherry, consistent with the selective *sfGFP* gene disruption. Figure 2b, panel II summarizes the fluorescence characteristics (*sfGFP*/mCherry) of the cells displayed in panel I, upon analyzing the integrated fluorescence intensities of three different frames of the respective fluorescence images corresponding to the four entries, demonstrating effective and selective disruption of the *sfGFP* gene. The disruption efficiency of the *sfGFP* gene by the LAC12aGE machinery is summarized in panel III, demonstrating a 67% disruption efficiency. A further experiment demonstrates the versatility of the LAC12aGE machinery to induce selective *sfGFP* gene disruption of the dual fluorescent reporter plasmid in HeLa cells (Figure S8). The light‐triggered LAC12aGE machinery stimulated selective disruption of the *sfGFP* gene in the exogenous dual fluorescent reporter plasmid shown in Figure 2a, further supported by light‐induced activation of a cell domain using a photomask, Figure 2c, panel I. A HEK293T cell culture loaded with the Cas12a plasmid and the caged OFF‐crRNA*_sfGFP_* was photochemically activated by irradiating the cell region with a photomask that separates the regions into two halves. The illuminated region activated the LAC12aGE machinery toward the *sfGFP* gene disruption process. Figure 2c, panel II shows the confocal fluorescence microscopy images demonstrating the selective activation of the LAC12GE machinery toward the disruption of the *sfGFP* gene in the cell region exposed to light for 5 min. In the absence of UV irradiation, regions i and ii show the fluorescence of *sfGFP* and mCherry, and there is no significant difference in the relative expression levels of *sfGFP* in the two regions (panel III). In the UV‐illuminated region iii, the green fluorescence of *sfGFP* is depleted while the red fluorescence of mCherry is observed, indicating light‐activated disruption of the *sfGFP* gene with a disruption efficiency of 69%. In comparison, the adjacent dark region iv demonstrates both fluorescence of *sfGFP* and mCherry. The difference in the expression levels of *sfGFP* between two regions is significant, demonstrating the spatial control over gene editing features of the LAC12aGE machinery.

### LAC12aGE‐Mediated Endogenous Gene Editing and Disruption in Cells

**Figure 3:**
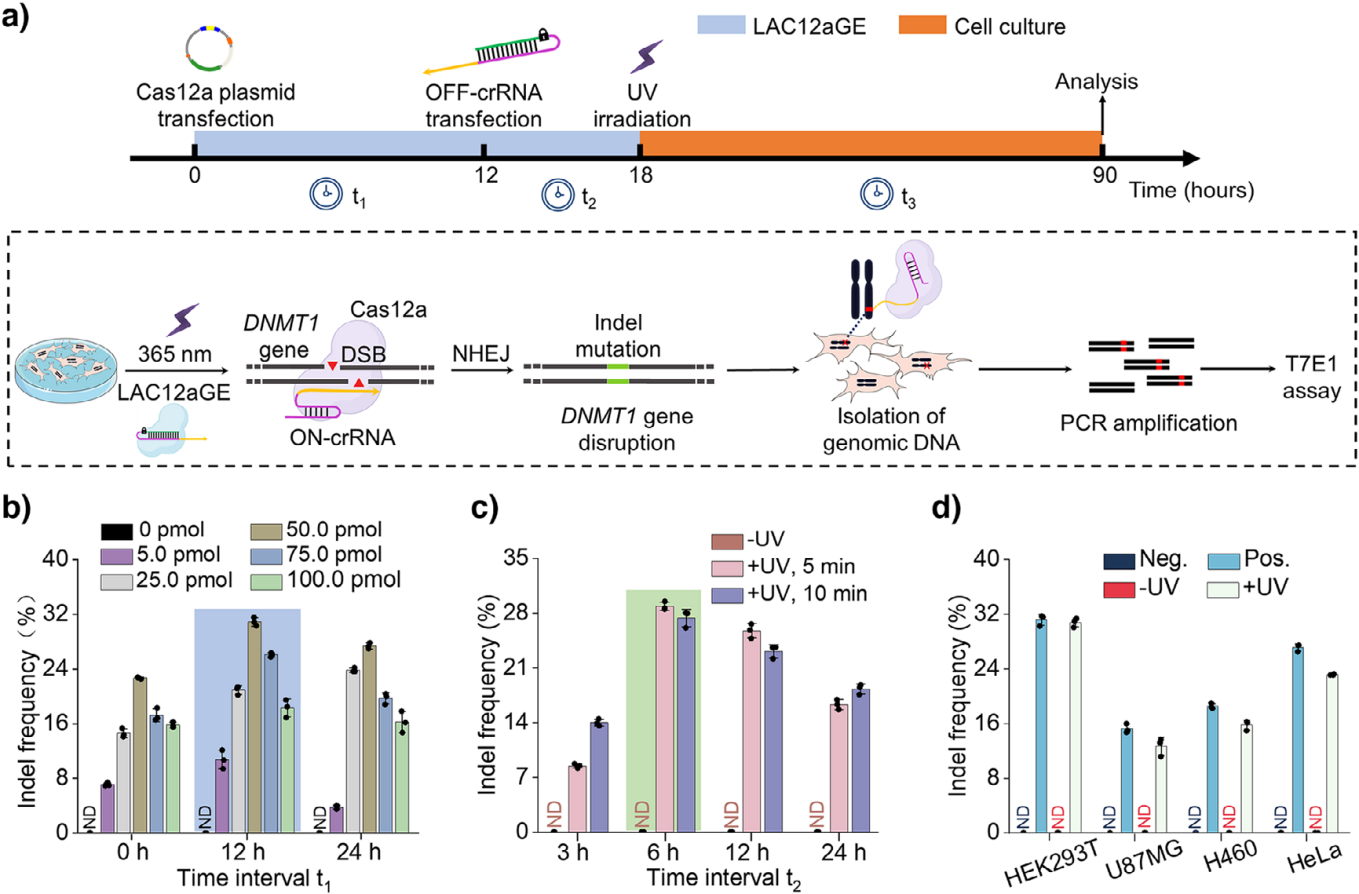
LAC12aGE machinery for endogenous gene editing and disruption. a) Schematic of temporal experimental steps implemented for the LAC12aGE‐mediated genome editing process. *t*_1_, Time interval between Cas12a plasmid transfection and OFF‐crRNA transfection. *t*_2_, Time interval between OFF‐crRNA transfection and UV illumination. *t*_3_, Time interval between UV illumination and indel analysis. The dashed box illustrates LAC12GE machinery‐mediated gene editing targeting the *DNMT1* gene, followed by disruption induced by NHEJ‐mediated indel mutations. The indel frequency is analyzed using a T7E1 assay after genomic DNA isolation and PCR amplification. b) Effects of variable time intervals of *t*_1_ and concentrations of OFF‐crRNA on the indel frequencies resulting from LAC12aGE‐mediated gene editing and disruption of *DNMT1* gene in HEK293T cells. c) Effects of variable time intervals of *t*_2_ and UV illumination on the indel frequencies resulting from LAC12aGE‐mediated gene editing and disruption of *DNMT1* gene in HEK293T cells. d) Indel frequencies of LAC12aGE‐mediated genome editing activities toward the *DNMT1* gene in HEK293T, U87MG, H460, and HeLa cells. Neg., negative control, transfected with Cas12a plasmid in the absence of crRNA*_DNMT1_*. Pos., positive control, transfected with Cas12a plasmid and wt‐crRNA*_DNMT1_*. −UV, cells transfected with Cas12a plasmid/OFF‐crRNA*_DNMT1_* in the absence of UV light. +UV, cells transfected with Cas12a plasmid/OFF‐crRNA*_DNMT1_* in the presence of UV light. Data are presented as mean ± SD (*n *= 3). The original gel electrophoresis experiments corresponding to the indel calculations are presented in Figures S12–S14.

The successful cellular application of the photo‐triggered LAC12aGE machinery for editing and disruption of the *sfGFP* gene in the exogenous dual fluorescent reporter plasmid was then extended to the LAC12aGE machinery for endogenous gene editing and disruption of the gene encoding DNA methyltransferase 1, i.e., *DNMT1* gene. *DNMT1* plays a key role in DNA methylation, ensuing inherited epigenetic patterns during replication.^[^ ^43^ ^]^ Mutation in *DNMT1* gene were identified to be linked with different disease,^[^ ^44^, ^45^ ^]^ and thus, editing and disruption of the *DNMT1* gene was suggested as a therapeutic means. We adopted the protocol outlined in Figure 3a to probe the efficacy of the LAC12aGE machinery toward editing and disrupting the *DNMT1* gene. At time *t* = 0, HEK293T cells were transfected with the Cas12a plasmid. At time *t* = 12 h, the cells were transfected with the photo‐responsive caged OFF‐crRNA*_DNMT1_*, and after a time interval of 6 h at *t* = 18 h, the cells were irradiated for 5 min (*λ* = 365 nm, 25 mW cm^−2^) to activate the LAC12aGE machinery. Subsequently, the cells were allowed to undergo the gene editing and disruption process for a time interval of 72 h. Then the cells were lysed, and the genomic DNA was isolated and amplified by polymerase chain reaction (PCR) (Table S3). Finally, the effectiveness of gene editing was evaluated using the T7E1 assay (Figure S9).^[^ ^46^ ^]^ This protocol involving HEK293T cells follow a set of experiments optimizing the pathway probing the editing process, as outlined in Figure 3b–d. Figure 3b displays the effects of concentrations of OFF‐crRNA*_DNMT1_* and the time‐intervals (*t*_1_) employed to transfect the cells with OFF‐crRNA*_DNMT1_* on the indel frequencies (editing efficacies). Evidently, at a concentration of 50 pmol of OFF‐crRNA*_DNMT1_* transfected after 12 h, optimal gene editing was observed (indel frequency 31%). The relatively low indel frequency might be attributed to the large size of the plasmide encoding the Cas12a protein (ca. 9 kbp), which could negatively impact transfection efficiency. To investigate the cell transfection efficiency, experiments were conducted using a Cas12a‐EGFP fusion plasmid. As shown in Figure S10, flow cytometry analysis of the HEK293T cells transfected with the Cas12a‐EGFP plasmid revealed a transfection efficiency of approximately 85%, supporting the notion that the large size of the Cas12a plasmid may indeed limit the transfection efficiency. Notably, at higher concentrations of OFF‐crRNA*_DNMT1_*, gene editing efficiency decreases. This phenomenon has been previously reported in other studies;^[^ ^16^, ^26^ ^]^ however, the underlying mechanism behind this inhibitory effect remains unclear. Figure 3c depicts the effects of time‐intervals (*t*_2_) and UV irradiation time employed to activate the Cas12a/OFF‐crRNA*_DNMT1_* machinery, demonstrating that after a time interval of 6 h and UV irradiation for 5 min, optimal editing efficacies were observed. Moreover, the results demonstrate that no editing proceeds without the light‐induced activation of the machinery. Furthermore, Figure S11 depicts the effect of irradiation time on the viability of the cells. Evidently, no damage to cell viability is observed in 15 min of irradiation under the used conditions. Thus, no damage to the cells occurs within the time intervals used to activate the LAC12aGE machinery. Figure 3d depicts the indel frequency (editing efficacy) of the *DNMT1* gene in a series of different cell lines. Evidently, effective gene disruptions are observed in HEK293T and HeLa cells. The original gel electrophoresis experiments corresponding to the indel calculations in Figures 3b–d are presented in Figures S12–S14. Further implementation of the LAC12aGE machinery to edit and disrupt the *VEGFA* gene in the HEK293T cell line are displayed in Figure S15.

### LAC12aGE‐Mediated Disruption of *HGF* Gene Promotes Cell Apoptosis

**Figure 4:**
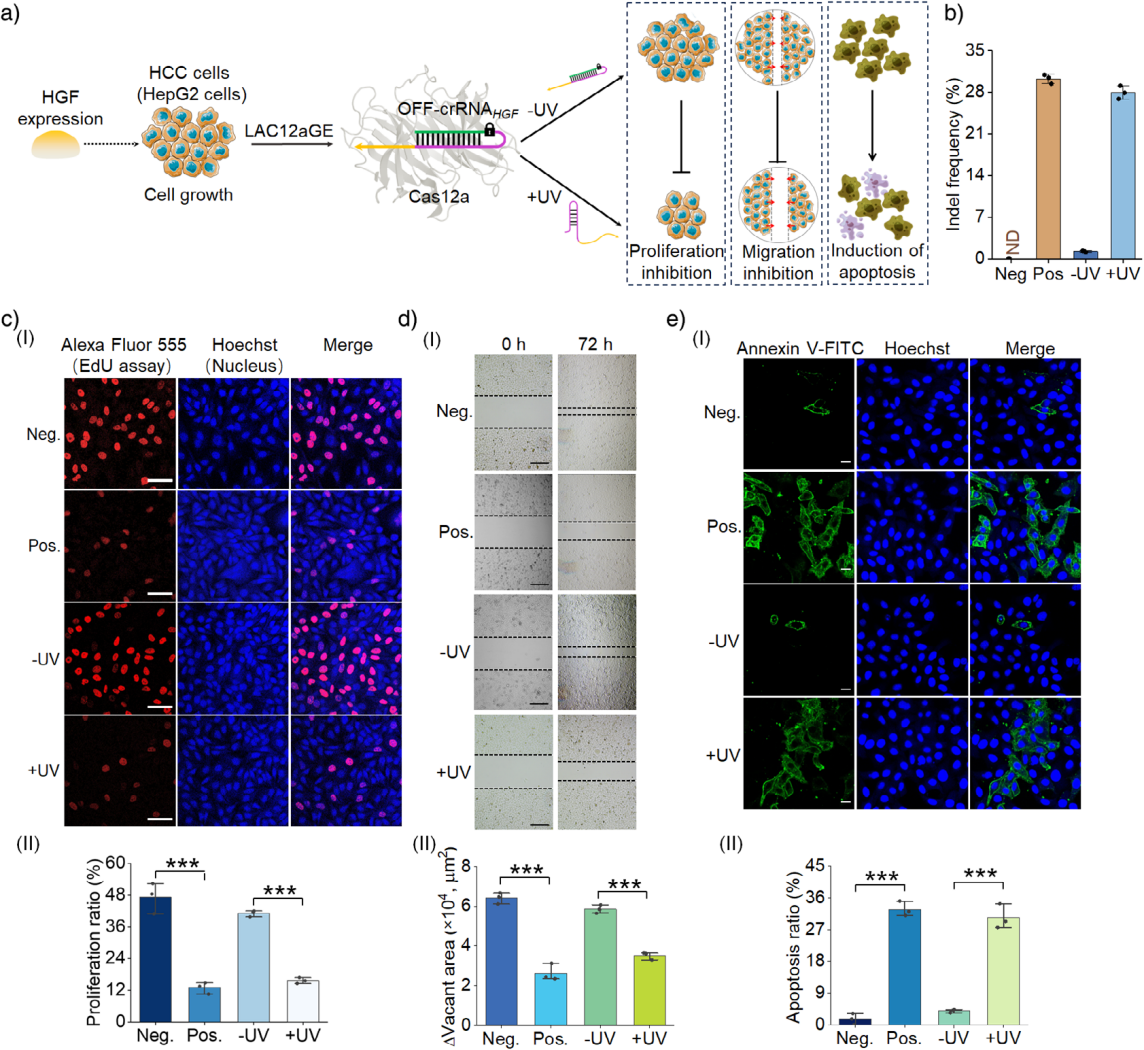
LAC12aGE‐mediated disruption of the *HGF* gene and its effects on cell proliferation, migration, and apoptosis. a) Schematic of LAC12aGE‐mediated disruption of the *HGF* gene in HepG2 cells exposed to UV light and the subsequent inhibited cell proliferation and migration, and induced apoptosis. b) Indel frequencies of LAC12aGE‐mediated genome editing targeting *HGF* gene in HepG2 cells in the absence or presence of UV illumination. The original gel electrophoresis experiments corresponding to the indel calculations are presented in Figures S16. Effects of variable LAC12aGE machineries on c) cell proliferation, d) migration, and e) apoptosis, including: Neg., negative control transfected with Cas12a plasmid in the absence of crRNA*_HGF_*; Pos., positive control transfected with Cas12a plasmid and wt‐crRNA*_HGF_*; −UV, cells transfected with Cas12a plasmid/OFF‐crRNA*_HGF_* in the absence of UV light; +UV, cells transfected with Cas12a plasmid/OFF‐crRNA*_HGF_* in the presence of UV light. c) Panel I: Confocal microscopy images of HepG2 cells stained with Alexa Fluor 555 (Edu assay) and Hoechst (nucleus) dyes. Scale bar, 50 µm. Panel II: Proliferation ratios of HepG2 cells corresponding to the experiments shown in panel I. The cell proliferation ratio (%) is defined as *m/n* × 100%, where *m* corresponds to the number of Alexa Fluor 555 red fluorescent cells, and *n* is the total number of Hoechst blue fluorescent cells. d) Panel I: Wound healing assay corresponding to cell migration capacities of HepG2 cells. Scale bar, 100 µm. Panel II: Changes in vacant area separating cells treated with the variable LAC12aGE configurations displayed in panel I. e) Panel I: Confocal microscopy images of HepG2 cells stained with Annexin V‐FITC (apoptosis evaluation) and Hoechst (nucleus) dyes. Scale bar, 20 µm. Panel II: Cell apoptosis ratios of HepG2 cells corresponding to the experiments shown in panel I. The cell apoptosis ratio (%) is defined as *x/y* × 100%, where *x* is the number of Annexin V‐positive apoptotic cells, and *y* is the total number of Hoechst blue fluorescent cells. ***, *P* < 0.001. Data are presented as mean ± SD (*n *= 3).

As one of the goals of the present study involves the application of the LAC12aGE machinery for in vivo gene editing and disruption of tumor growth‐related factors, we attempted to examine the application of the LAC12aGE machinery for in vitro gene editing and disruption of the *HGF* gene. *HGF* is a multifunctional growth factor generated by hepatocytes and other cells, playing key roles in hepatocellular carcinoma (HCC) cells.^[^ ^47^ ^]^ The association of *HGF* with the c‐Met receptor mediates the downstream signaling pathways promoting cell proliferation and migration.^[^ ^48^ ^]^ Thus, disrupting the *HGF* gene promotes cell apoptosis.^[^ ^49^ ^]^ Accordingly, we applied the LAC12aGE machinery to edit and disrupt the *HGF* gene associated with hepatocellular HepG2, following the protocol described in Figure 3a. The path for the in vitro disruption of the *HGF* gene in HCC cells is schematically presented in Figure 4a. The HepG2 cells were subjected to the Cas12a plasmid transfection, followed by transfection with the OFF‐crRNA*_HGF_* to develop the intracellular inactive LAC12aGE machinery. The cells were then subjected to UV‐triggered activation of the LAC12aGE machinery, anticipated to disrupt the gene through indel mutations, leading to inhibition of cell proliferation, migration, and enhanced cell apoptosis. These cellular events were then quantitatively analyzed and compared to control systems that included a negative control involving the Cas12a plasmid in the absence of crRNA*_HGF_* and positive control cells transfected with the intact Cas12a/crRNA*_HGF_* with no need of light‐triggered activation. Figure 4b depicts the analysis of indel frequency evaluated in the light‐activated, LAC12aGE machinery‐transfected cells, in comparison to the non‐illuminated cells containing the LAC12aGE machinery. Evidently, effective gene editing was observed in the light‐activated cells (indel frequency 28%). (The original gel electrophoresis experiments corresponding to the indel calculations in Figure 4b are presented in Figures S16.) Figure 4c, panel I depicts the confocal fluorescence microscopy images corresponding to the proliferation efficacities in the LAC12aGE machinery‐loaded HepG2 cells and control systems, using the Edu fluorescent assay. Effective inhibition of cell proliferation was observed in cells treated with the Cas12a/crRNA*_HGF_* machinery or the light‐activated cells loaded with the Cas12a/OFF‐crRNA*_HGF_* machinery. In contrast, effective proliferation was observed in the cells loaded with the Cas12a plasmid only or in the non‐light‐ illuminated cells loaded with Cas12a/OFF‐crRNA*_HGF_* machinery.

Figure 4c, panel II shows the statistical quantitative proliferation ratio (the percentage of EdU red fluorescence‐positive proliferating cells divided by the total number of Hoechst blue fluorescence‐positive cells) for the HepG2 cells treated with the light‐activated Cas12a/OFF‐crRNA*_HGF_* machinery, in comparison to the control systems. The results demonstrate effective inhibition of HepG2 cell proliferation by the light‐activated Cas12a/OFF‐crRNA*_HGF_* gene editing. Figure 4d, panel I probes the migration characteristics of the light‐triggered Cas12a/OFF‐crRNA*_HGF_*‐loaded HepG2 cells after gene editing and disruption for a time interval of 72 h, in comparison to control systems. The migration features of the cells were evaluated by measuring their ability to bridge a scratched gap separating the cells. While the gap was significantly occupied by the negative control cells loaded with only the Cas12a plasmid or the non‐light‐activated Cas12a/OFF‐crRNA*_HGF_*, implying effective migration features. In contrast, the positive control cells loaded with Cas12a/crRNA*_HGF_*, as well as the light‐activated Cas12a/OFF‐crRNA*_HGF_*‐loaded cells, revealed marked inhibition of migration toward bridging the scratched gap. Figure 4d, panel II summarizes the empty areas of non‐occupied scratched domains corresponding to the light‐ activated Cas12a/OFF‐crRNA*_HGF_*‐loaded cells and control systems, based on the analysis of three different experiments. Evidently, the light‐activated gene‐edited cells demonstrate inhibited migration features, consistent with the disruption of the *HGF* gene. Figure 4e depicts the confocal microscopy images probing the degree of apoptosis in the light‐activated Cas12a/OFF‐crRNA*_HGF_*‐loaded HepG2 cells compared to the control systems. Apoptosis was evaluated by staining the cells with Annexin V‐FITC (green fluorescence), which accumulates in apoptotic cells, and using the blue Hoechst reagent (blue fluorescence) to quantify the total number of cells. The apoptosis efficiency was quantified by calculating the apoptosis ratio (%) of Annexin V green fluorescence‐positive apoptotic cells divided by the total number of Hoechst blue fluorescence‐positive cells. The negative control consisting of Cas12a plasmid‐loaded cells (without crRNA*_HGF_*) or the non‐light‐activated Cas12a/OFF‐crRNA*_HGF_*‐loaded cells revealed low levels of apoptosis, consistent with efficient expression of the *HGF* gene. Conversely, the positive control, consisting of the Cas12a/crRNA*_HGF_*‐loaded HepG2 cells or the light‐activated Cas12a/OFF‐crRNA*_HGF_*‐loaded HepG2 cells, showed high levels of apoptosis, confirming the disruption of the *HGF* gene by the light‐activated CRISPR machinery. Figure 4e, panel II summarizes the apoptosis ratio observed in the light‐activated Cas12a/OFF‐crRNA*_HGF_*‐loaded HepG2 cells and control systems, based on three independent experiments. The results demonstrate the effective disruption of the *HGF* gene by the LAC12aGE machinery, leading to effective apoptosis of the cells. In addition, experiments probing the *HGF* gene editing and disruption in Hep3B cells are presented in Figures S17–S20. For additional flow cytometry experiments supporting *HGF* gene editing‐induced apoptosis in HepG2 and Hep3B cells, see Figure S21 and the accompanying discussion. Moreover, the LAC12aGE machinery‐induced *HGF* gene editing process was further validated by probing the relative expression levels of the *HGF* gene in HepG2 and Hep3B cells using reverse transcription‐quantitative PCR (RT‐qPCR) analysis (Figure S22 and Table S4). The results were consistent with the indel frequency analysis obtained from the T7E1 assay, as shown in Figures 4b and S17b.

**Figure 5:**
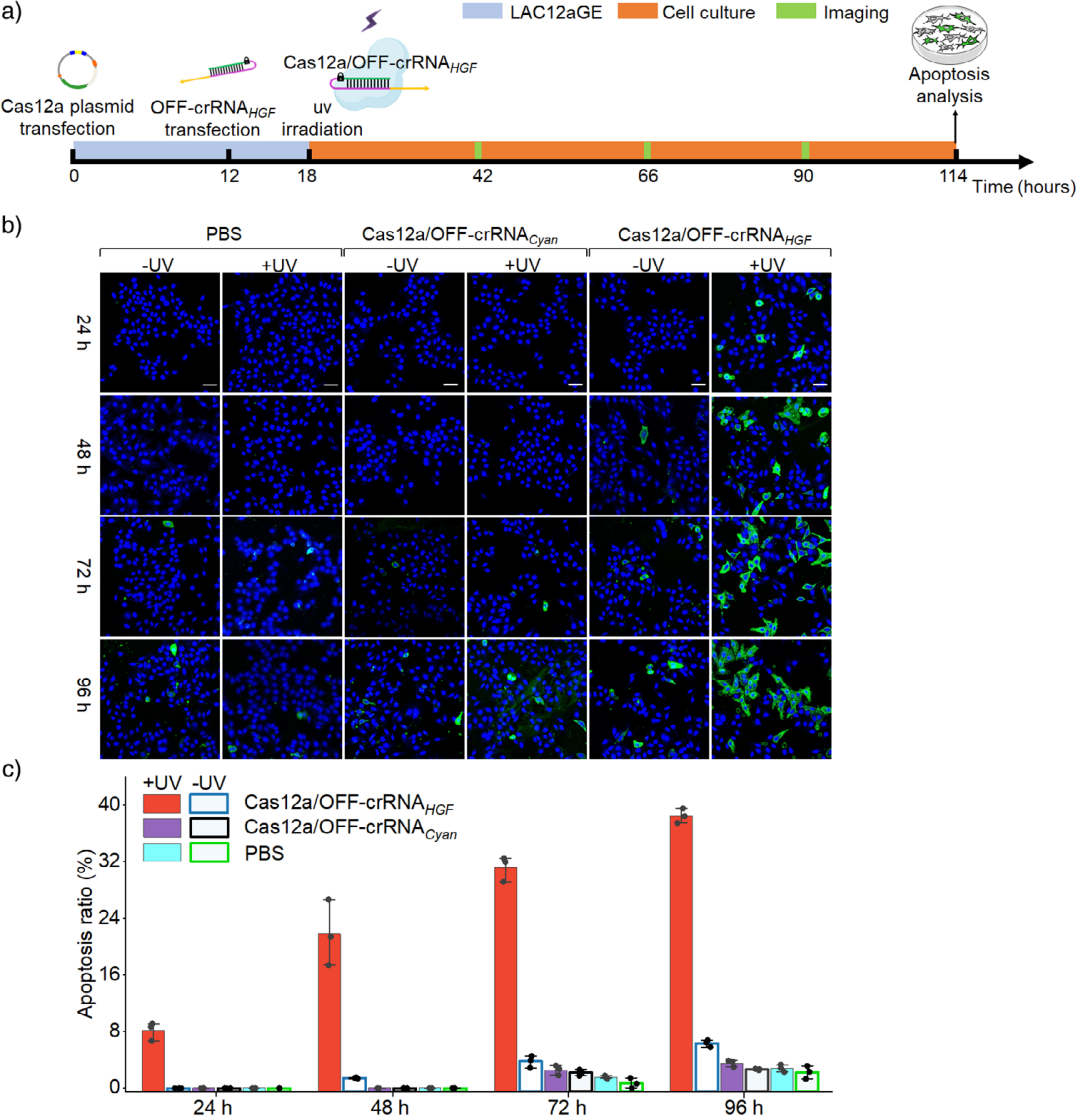
Dynamic apoptosis induced by LAC12aGE‐mediated gene editing and disruption of *HGF* gene in HepG2 cells. a) Schematic of the stepwise protocol evaluating the temporal apoptosis induced by LAC12aGE‐mediated gene editing and disruption of *HGF* gene in HepG2 cells. b) Confocal microscopy images depicting the temporal apoptosis of HepG2 cells stained with Annexin V‐FITC (apoptosis) and Hoechst (nucleus) dyes in different groups treated with Cas12a/OFF‐crRNA*_HGF_*, Cas12a/OFF‐crRNA*_Cyan_*, or PBS, in the presence or absence of UV illumination. Scale bar, 50 µm. c) Cell apoptosis ratios of HepG2 cells corresponding to the experiments shown in (b). Data are presented as mean ± SD (*n *= 3).

Moreover, the dynamics of the *HGF* gene editing and disruption by the light‐activated Cas12a/OFF‐crRNA*_HGF_* machinery and the associated apoptosis of HepG2 cells were evaluated, Figure 5a. In these experiments, HepG2 cells were transfected with the Cas12a plasmid and OFF‐crRNA*_HGF_*, followed by the formation of the intracellular LAC12aGE machinery for 18 h. The intracellular LAC12aGE machinery was then activated by light (*λ* = 365 nm, 25 mW cm^−2^) for 5 min, and the temporal knockout of *HGF* gene by the CRISPR machinery was evaluated by monitoring the dynamic apoptosis features of the cells at various time intervals of gene editing. Three groups of HepG2 cells were examined in this study. The first group included HepG2 cells lacking the CRISPR machinery but treated or untreated with UV light. The second group was transfected with Cas12a plasmid and unmatched OFF‐crRNA (e.g., OFF‐crRNA*_Cyan_*), and subjected to the light activation or tested under dark conditions. The third group was transfected with the Cas12a/OFF‐crRNA*_HGF_* machinery and activated by light or subjected to dark conditions. The temporal apoptosis demonstrated by these three groups was evaluated at time intervals using the Annexin V‐FITC (green) and Hoechst (blue) staining assay, over a time period of 96 h, Figure 5b. Only the light‐activated HepG2 cells transfected with the Cas12a/OFF‐crRNA*_HGF_* revealed visible apoptosis. Figure 5c summarizes the time‐dependent apoptosis ratios (%) for the different groups displayed in Figure 5b, based on statistics from three independent experiments. The apoptosis ratios increased as the time interval of the LAC12aGE (crRNA*_HGF_*) machinery was extended, consistent with the progressive editing and disruption of the *HGF* gene over time.

### LAC12aGE‐Mediated Disruption of *HGF* Gene for Tumor Repression in Mice

**Figure 6:**
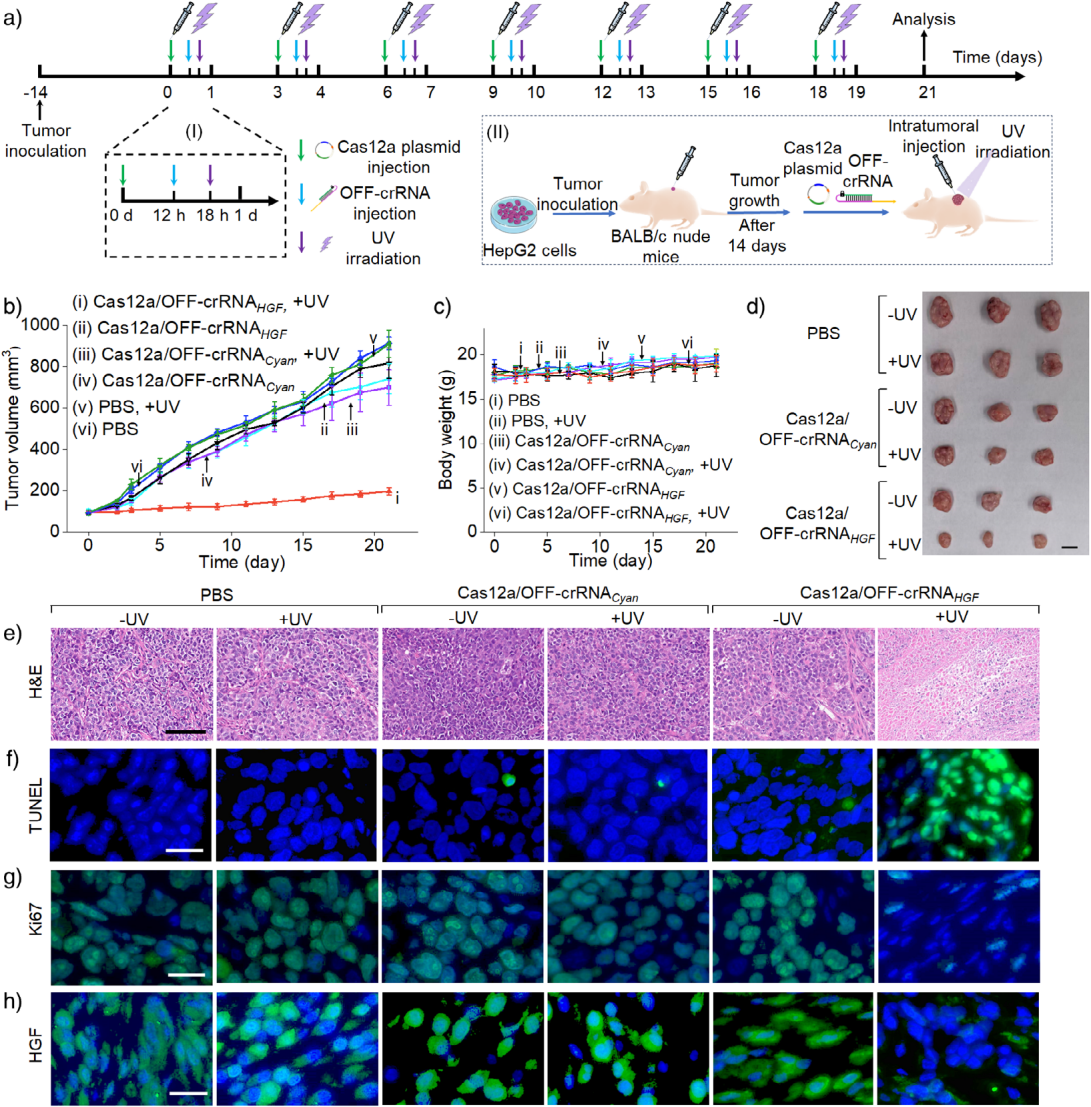
LAC12aGE‐mediated disruption of the *HGF* gene resulting in tumor repression in Xenograft BALB/c nude mice bearing HepG2 tumors. a) Schematic timeline and protocol corresponding to the in vivo light‐triggered Cas12a/crRNA machinery gene editing process. Panel I: Timeline of the LAC12aGE machinery treatment within a single cycle. Panel II: Schematic of the procedure for HepG2 tumor inoculation in BALB/c nude mice and intratumoral injection of LAC12aGE machinery for light‐activated in vivo gene editing. UV illumiantion: 365 nm, 40 mW cm^−2^, 10 min. b) Temporal changes of the tumor volumes in mice treated with (i) Cas12a/OFF‐crRNA*_HGF_* in the presence of UV light, (ii) Cas12a/OFF‐crRNA*_HGF_* in the absence of UV light, (iii) Cas12a/OFF‐crRNA*_Cyan_* in the presence of UV light, (iv) Cas12a/OFF‐crRNA*_Cyan_* in the absence of UV light, (v) PBS in the presence of UV light, and (vi) PBS in the absence of UV light. Note: PBS solution contained the jetPEI transfection reagent. c) Changes in body weight of mice treated with (i) PBS in the absence of UV light, (ii) PBS in the presence of UV light, (iii) Cas12a/OFF‐crRNA*_Cyan_* in the absence of UV light, (iv) Cas12a/OFF‐crRNA*_Cyan_* in the presence of UV light, (v) Cas12a/OFF‐crRNA*_HGF_* in the absence of UV light, and (vi) Cas12a/OFF‐crRNA*_HGF_* in the presence of UV light. d) Representative images of the tumors excised from the mice treated with the light‐activated Cas12a/OFF‐crRNA*_HGF_* (bottom entry) and the control systems. Scale bar, 1 cm. Data are presented as mean ± SD (*n *= 3). e)‒h) Representative microscopy images of the tumor tissues from the mice treated with PBS, Cas12a/OFF‐crRNA*_Cyan_*, or Cas12a/OFF‐crRNA*_HGF_*, in the absence or presence of UV illumination, showing the levels of cell necrosis, apoptosis, proliferation‐associated Ki67 expression, and *HGF* expression, respectively. e) Hematoxylin and eosin (H&E) staining of tumor tissues revealing cell necrosis levels in different groups of mice. Scale bars, 100 µm. f) Terminal deoxynucleotidyl transferase–mediated deoxyuridine triphosphate nick end labeling (TUNEL) staining of tumor tissues revealing cell apoptosis levels in different groups of mice. Scale bars, 20 µm. g) Immunofluorescence staining assay probing Ki67 nuclear protein expression levels in tumor tissues from different groups of mice. Scale bars, 20 µm. h) Immunofluorescence staining assay probing *HGF* protein expression levels in tumor tissues from different groups of mice. Scale bars, 20 µm. (For quantitative evaluation of the fluorescence intensities in different samples, see Figure S27.).

In the next step, the light‐stimulated CRISPR gene editing machinery was applied for in vivo knockout and disruption of the *HGF* gene in HepG2 tumors. Figure 6a depicts the protocol of the in vivo light‐activated gene editing process. HepG2 cell tumors were inoculated in Xenograft BALB/c nude mice for a time interval of 14 days, and subsequently subjected to the intratumoral transfection with the Cas12a plasmid and the OFF‐crRNA*_HGF_*. The transfected tumors were subjected to the light‐triggered activation of the Cas12a/OFF‐crRNA*_HGF_* machinery (*λ* = 365 nm, 40 mW cm^−2^, 10 min), followed by the gene editing process for a time interval of 3 days. (For an experiment demonstrating the penetration ability of UV light through mouse skin, see Figure S23 and the accompanying discussion. For experimental validation of light‐induced photodeprotection of the OFF‐crRNA*_HGF_* within the mouse tumor, an essential step for initiating gene editing in tumors, refer to Figure S24 and the accompanying discussion.) The transfection of the Cas12a plasmid/OFF‐crRNA*_HGF_* and light activation process was repeated over seven cycles, resulting in a total treatment duration of 21 days. The detailed temporal progression of the LAC12aGE machinery within one cycle is displayed in panel I. Appropriate control tumors were prepared following the same procedure. The gene editing efficiencies were evaluated by tracking the time‐dependent tumor sizes during treatment, measuring the excised tumor sizes, assessing apoptotic levels, and conducting histological characterization of the treated tumor cells. Figure 6b, curve i depicts the time‐dependent sizes of the tumors in mice treated with the light‐activated Cas12a/OFF‐crRNA*_HGF_* machinery. The results show minimal tumor growth, from 100 to 200 mm^3^ over the 21 day treatment period. In contrast, the time‐dependent growth of tumors in the control groups, including (ii) Cas12a/OFF‐crRNA*_HGF_* without UV light, (iii) light‐activated Cas12a/OFF‐crRNA*_Cyan_* (a foreign/unmatched crRNA), (iv) Cas12a/OFF‐crRNA*_Cyan_* without UV light, (v) phosphate‐buffered saline (PBS) with UV light, and (vi) PBS without UV light, exhibit significant tumor growth over the 21 day period, with tumor sizes increasing from 100 to 700–900 mm^3^. These results demonstrate that tumors treated with the light‐activated Cas12a/OFF‐crRNA*_HGF_* gene editing machinery undergo effective knockout and disruption of the *HGF* gene, as reflected by very little size changes of the tumors. In contrast, the rapid tumor growth in all control systems is consistent with the lack of any gene disruption in the absence of light activation and matched crRNAs. It should be noted that the PBS control group received in vivo jetPEI alone, without the LAC12aE components. The absence of observable tumor growth inhibition in this group indicates that the transfection reagent alone does not contribute to antitumor effects during the treatment process. This highlights the specificity and precision of the gene editing process. Figure 6c depicts the changes in mice weight over the 21 day treatment period of the tumors with different Cas12a/OFF‐crRNA machineries or PBS, both with and without UV light activation. No significant weight changes are observed in any of the groups. Particularly, there are no weight changes in the mice bearing tumors subjected to the light‐activated Cas12a/OFF‐crRNA*_HGF_* machinery, indicating that the gene editing machinery and UV light have no damaging effect on the mice. Figure 6d shows representative images of the tumors excised from the mice treated with the light‐activated Cas12a/OFF‐crRNA*_HGF_* (bottom entry) and the control tumor systems. While the tumors subjected to the light‐activated Cas12a/OFF‐crRNA*_HGF_* reveal a volume of ca. 200 mm^3^ at 21 days, the control systems demonstrate substantially large tumor volumes in the range of 700–910 mm^3^. The indel frequencies resulting from gene editing in HepG2 tumors using the light‐activated Cas12a/OFF‐crRNA*_HGF_* machinery and appropriate controls are depicted in Figure S25. In these experiments, the LAC12aGE machinery or control constructs were transfected into mouse tumors. After a time interval of 72 h, the tumors were excised, dissociated into single‐cell suspensions, and subjected to T7E1 assay. The resulting samples were analyzed via quantitative electrophoresis to evaluate indel frequencies. An indel frequency of approximately 18% was observed, indicating moderate gene‐editing efficiency in vivo. To further evaluate the impact of plasmid size on transfection efficiency, a Cas12a‐EGFP fusion plasmid was transfected into HepG2 tumors. After 72 h, the tumors were harvested, dissociated into single‐cell suspensions, and analyzed by flow cytometry. The results in Figure S26 revealed a transfection efficiency of ca. 52%, demonstrating that the Cas12a plasmid can be transfected into HepG2 tumors, despite its relatively large size. Figure 6e–h shows representative microscopy images of the tumor tissues from the mice treated with the light‐activated Cas12a/OFF‐crRNA*_HGF_* or the control systems, probing cell apoptosis/necrosis and other related cellular functional parameters. In Figure 6e, the necrosis of the tumor tissue treated with the light‐activated Cas12a/OFF‐crRNA*_HGF_* is compared to that in the control systems using the hematoxylin and eosin (H&E) staining assay. In the light‐activated Cas12a/OFF‐crRNA*_HGF_*‐treated tumors, the blue‐stained nuclei disappear, while red cytoplasm staining is observed, indicating a high necrosis level in the tumor tissue. Figure 6f probes the apoptosis of tumor tissue using the TUNEl staining (terminal deoxynucleotidyl transferase‐mediated deoxyuridine triphosphate nick end labeling) procedure. The tumors treated with the light‐activated Cas12a/OFF‐crRNA*_HGF_* exhibit high levels of apoptosis, as reflected by the green fluorescence of FITC‐labeled dUTP, while the control systems show low levels of apoptosis. Figure 6g show the immunofluorescence staining assay probing the expression levels of Ki67 nuclear protein and related proliferation efficacies. A depletion of the green fluorescence associated with proliferating cells is observed in the light‐activated Cas12a/OFF‐crRNA*_HGF_*‐treated tumors compared to the control systems, consistent with the inhibition of proliferation process. Figure 6h depicts *HGF* expression levels using the immunofluorescence staining assay. The *HGF* is effectively expressed in all control systems, while the light‐activated, Cas12a/OFF‐crRNA*_HGF_*‐treated tumors show low levels of *HGF* expression, consistent with the disruption of *HGF* gene. Quantitative analysis of the fluorescence intensity of TUNEL (Figure 6f), Ki67 (Figure 6g), and *HGF* immunofluorescence staining (Figure 6h) in tumor tissues for the different groups of mice are presented in Figure S27. Further histological analysis probing different organs of the HepG2 tumor‐bearing mice treated with light‐triggered Cas12a/OFF‐crRNA*_HGF_* and control systems is presented in Figure S28.

## Conclusion

The present study introduced a versatile photo‐triggered CRISPR/Cas12a machinery for in vitro and in vivo gene editing. The machinery involves a single photo‐responsive *ortho*‐nitrobenzyl phosphate ester‐caging unit, which is photochemically unlocked toward initiating the gene editing process. Spatiotemporal, leakage‐free, exogenous, and endogenous gene editing by the LAC12aGE machinery were demonstrated. The light‐activated disruption efficiency of the exogenous *sfGFP* gene using LAC12aGE machinery (69%) is comparable to the previously reported exogenous gene disruption efficiency using CRISPR/Cas12a system.^[^ ^50^ ^]^ These include the light‐stimulated disruption of the *DNMT1* gene in HEK293T cells, and the knockout of the *HGF* gene in HCC cells. The indel frequency demonstrated by the light‐stimulated disruption of the *DNMT1* or *HGF* gene in cells corresponded to ca. 28% — a value slightly higher than the indel frequency reported for *DNMT1* gene disruption in HEK293T cells using the CRISPR/Cas12a system,^[^ ^3^ ^]^ which lacks the spatiotemporal advantages of the LAC12aGE machinery. Moreover, the LAC12aGE machinery was successfully applied for the effective in vivo induction of apoptosis and repression of HepG2 tumors in mice. Thus, the LAC12aGE machinery provides promising advantages for precise gene editing in vivo, with potential for diverse therapeutic uses.

Beyond the versatile gene‐editing capabilities of the LAC12aGE machinery for future therapeutics, several challenges remain in its development. Improving the transport and transfection efficiencies of the CRISPR machinery into cells is certainly an interesting path to explore. The integration of the LAC12aGE machinery within biomarker‐responsive carriers, such as hydrogel microcapsules or metal‐organic framework nanoparticles,^[^ ^51^, ^52^ ^]^ and the modification of the carriers with targeting aptamers and/or DNA tetrahedra nanostructures as cell permeation agents, could improve the targeted selectivity and transfection efficacy. In addition, the current LAC12aGE machinery is activated by UV light (*λ* = 365 nm) that might be harmful to biological tissues and poses limitations for applications in deep organs. Chemical modification of the photo‐responsive protecting groups or the use of alternative photo‐responsive caging agents,^[^ ^19^ ^]^ and employing upconversion nanoparticles with infrared illuminations,^[^ ^39^ ^]^ could resolve these limitations and eventually enable the development of light‐controlled, multiple gene editing platforms. Furthermore, extending the LAC12aGE machinery for editing genes associated with other diseases is a desirable goal.

## Conflict of Interests

The authors declare no conflict of interest.

## Supporting information



Supporting Information

## Data Availability

The data that support the findings of this study are available from the corresponding author upon reasonable request.

## References

[1] J. D. Sander , J. K. Joung , Nat. Biotechnol. 2014, 32, 347–355. 24584096 10.1038/nbt.2842 PMC4022601

[2] P. D. Hsu , E. S. Lander , F. Zhang , Cell 2014, 157, 1262–1278. 24906146 10.1016/j.cell.2014.05.010 PMC4343198

[3] B. Zetsche , J. S. Gootenberg , O. O. Abudayyeh , I. M. Slaymaker , K. S. Makarova , P. Essletzbichler , S. E. Volz , J. Joung , J. van der Oost , A. Regev , E. V. Koonin , F. Zhang , Cell 2015, 163, 759–771. 26422227 10.1016/j.cell.2015.09.038 PMC4638220

[4] B. Li , W. Zhao , X. Luo , X. Zhang , C. Li , C. Zeng , Y. Dong , Nat. Biomed. Eng. 2017, 1, 0066. 28840077 10.1038/s41551-017-0066 PMC5562407

[5] G. Liu , Q. Lin , S. Jin , C. Gao , Mol. Cell 2022, 82, 333–347. 34968414 10.1016/j.molcel.2021.12.002

[6] R. Cai , R. Lv , X. Shi , G. Yang , J. Jin , Int. J. Mol. Sci. 2023, 24, 14865. 37834313 10.3390/ijms241914865 PMC10573330

[7] T. Li , Y. Yang , H. Qi , W. Cui , L. Zhang , X. Fu , X. He , M. Liu , P.‐f. Li , T. Yu , Signal Transduct. Target. Ther. 2023, 8, 36. 36646687 10.1038/s41392-023-01309-7 PMC9841506

[8] M. Chavez , X. Chen , P. B. Finn , L. S. Qi , Nat. Rev. Nephrol. 2023, 19, 9–22. 36280707 10.1038/s41581-022-00636-2 PMC9589773

[9] X. Zuo , C. Fan , H.‐Y. Chen , Nat. Biomed. Eng. 2017, 1, 0091.

[10] M. M. Kaminski , O. O. Abudayyeh , J. S. Gootenberg , F. Zhang , J. J. Collins , Nat. Biomed. Eng. 2021, 5, 643–656. 34272525 10.1038/s41551-021-00760-7

[11] B. P. Kleinstiver , A. A. Sousa , R. T. Walton , Y. E. Tak , J. Y. Hsu , K. Clement , M. M. Welch , J. E. Horng , J. Malagon‐Lopez , I. Scarfò , M. V. Maus , L. Pinello , M. J. Aryee , J. K. Joung , Nat. Biotechnol. 2019, 37, 276–282. 30742127 10.1038/s41587-018-0011-0 PMC6401248

[12] B. Zetsche , M. Heidenreich , P. Mohanraju , I. Fedorova , J. Kneppers , E. M. DeGennaro , N. Winblad , S. R. Choudhury , O. O. Abudayyeh , J. S. Gootenberg , W. Y. Wu , D. A. Scott , K. Severinov , J. van der Oost , F. Zhang , Nat. Biotechnol. 2017, 35, 31–34. 27918548 10.1038/nbt.3737 PMC5225075

[13] X. Ling , L. Chang , H. Chen , X. Gao , J. Yin , Y. Zuo , Y. Huang , B. Zhang , J. Hu , T. Liu , Mol. Cell 2021, 81, 4747–4756.e7. 34648747 10.1016/j.molcel.2021.09.021

[14] M. Breinig , A. Y. Schweitzer , A. M. Herianto , S. Revia , L. Schaefer , L. Wendler , A. Cobos Galvez , D. F. Tschaharganeh , Nat. Methods 2019, 16, 51–54. 30559432 10.1038/s41592-018-0262-1

[15] D. Kim , J. Kim , J. K. Hur , K. W. Been , S.‐H. Yoon , J.‐S. Kim , Nat. Biotechnol. 2016, 34, 863–868. 27272384 10.1038/nbt.3609

[16] J. K. Hur , K. Kim , K. W. Been , G. Baek , S. Ye , J. W. Hur , S. M. Ryu , Y. S. Lee , J. S. Kim , Nat. Biotechnol. 2016, 34, 807–808. 27272385 10.1038/nbt.3596

[17] W. Zhou , A. Deiters , Angew. Chem. Int. Ed. 2016, 128, 5394–5399; 10.1002/anie.201511441 26996256

[18] N. Ankenbruck , T. Courtney , Y. Naro , A. Deiters , Angew. Chem. Int. Ed. 2018, 130, 2768–2798; 10.1002/anie.201700171 PMC6026863 28521066

[19] M. P. O'Hagan , Z. Duan , F. Huang , S. Laps , J. Dong , F. Xia , I. Willner , Chem. Rev. 2023, 123, 6839–6887. 37078690 10.1021/acs.chemrev.3c00016 PMC10214457

[20] J. Wang , Z. Li , I. Willner , Angew. Chem. Int. Ed. 2023, 62, e202215332; 10.1002/anie.202215332 36651472

[21] Y. Liu , R. S. Zou , S. He , Y. Nihongaki , X. Li , S. Razavi , B. Wu , T. Ha , Science 2020, 368, 1265–1269. 32527834 10.1126/science.aay8204 PMC7608738

[22] W. Zhou , W. Brown , A. Bardhan , M. Delaney , A. S. Ilk , R. R. Rauen , S. I. Kahn , M. Tsang , A. Deiters , Angew. Chem. Int. Ed. 2020, 132, 8998–9003; 10.1002/anie.201914575 PMC7250724 32160370

[23] E. V. Moroz‐Omori , D. Satyapertiwi , M.‐C. Ramel , H. Høgset , I. K. Sunyovszki , Z. Liu , J. P. Wojciechowski , Y. Zhang , C. L. Grigsby , L. Brito , L. Bugeon , M. J. Dallman , M. M. Stevens , ACS Cent. Sci. 2020, 6, 695–703. 32490186 10.1021/acscentsci.9b01093 PMC7256956

[24] Y. Zhang , X. Ling , X. Su , S. Zhang , J. Wang , P. Zhang , W. Feng , Y. Y. Zhu , T. Liu , X. Tang , Angew. Chem. Int. Ed. 2020, 132, 20895–20899; 10.1002/anie.202009890 33448579

[25] D. Zhang , L. Liu , S. Jin , E. Tota , Z. Li , X. Piao , X. Zhang , X.‐D. Fu , N. K. Devaraj , J. Am. Chem. Soc. 2022, 144, 4487–4495. 35257575 10.1021/jacs.1c12166 PMC9469474

[26] Y.‐J. Sun , W.‐D. Chen , J. Liu , J.‐J. Li , Y. Zhang , W.‐Q. Cai , L. Liu , X.‐J. Tang , J. Hou , M. Wang , L. Cheng , Angew. Chem. Int. Ed. 2023, 62, e202212413; 10.1002/anie.202212413 36453982

[27] P. K. Jain , V. Ramanan , A. G. Schepers , N. S. Dalvie , A. Panda , H. E. Fleming , S. N. Bhatia , Angew. Chem. Int. Ed. 2016, 128, 12440–12444; 10.1002/anie.201606123 PMC5864249 27554600

[28] J. Wu , H. Peng , X. Lu , M. Lai , H. Zhang , X. C. Le , Angew. Chem. Int. Ed. 2021, 133, 11104–11109; 10.1002/anie.202014162 PMC8252003 33354860

[29] Y. Nihongaki , F. Kawano , T. Nakajima , M. Sato , Nat. Biotechnol. 2015, 33, 755–760. 26076431 10.1038/nbt.3245

[30] T. Takao , M. Sato , T. Maruyama , Proc. Natl. Acad. Sci. USA 2020, 117, 28579–28581. 33139551 10.1073/pnas.2016850117 PMC7682590

[31] Y. Nihongaki , T. Otabe , Y. Ueda , M. Sato , Nat. Chem. Biol. 2019, 15, 882–888. 31406371 10.1038/s41589-019-0338-y

[32] Y. Yu , X. Wu , N. Guan , J. Shao , H. Li , Y. Chen , Y. Ping , D. Li , H. Ye , Sci. Adv. 2020, 6, eabb1777. 32923591 10.1126/sciadv.abb1777 PMC7455487

[33] X. Wang , K. Dong , D. Kong , Y. Zhou , J. Yin , F. Cai , M. Wang , H. Ye , Sci. Adv. 2021, 7, eabh2358. 34890237 10.1126/sciadv.abh2358 PMC8664267

[34] C. Zhuo , J. Zhang , J.‐H. Lee , J. Jiao , D. Cheng , L. Liu , H.‐W. Kim , Y. Tao , M. Li , Signal Transduct. Target. Ther. 2021, 6, 238. 34148061 10.1038/s41392-021-00645-w PMC8214627

[35] M. Hu , Z. Qiu , Z. Bi , T. Tian , Y. Jiang , X. Zhou , Proc. Natl. Acad. Sci. USA 2022, 119, e2202034119. 35727982 10.1073/pnas.2202034119 PMC9245704

[36] H. Liu , J. Dong , Z. Duan , F. Xia , I. Willner , F. Huang , Sci. Adv. 2024, 10, eadp6166. 39047109 10.1126/sciadv.adp6166 PMC11268419

[37] M. Hu , R. Liu , Z. Qiu , F. Cao , T. Tian , Y. Lu , Y. Jiang , X. Zhou , Angew. Chem. Int. Ed. 2023, 62, e202300663; 10.1002/anie.202300663 37016515

[38] P. Liu , Y. Lin , X. Zhuo , J. Zeng , B. Chen , Z. Zou , G. Liu , E. Xiong , R. Yang , Angew. Chem. Int. Ed. 2024, 63, e202401486; 10.1002/anie.202401486 38563640

[39] Y. Pan , J. Yang , X. Luan , X. Liu , X. Li , J. Yang , T. Huang , L. Sun , Y. Wang , Y. Lin , Y. Song , Sci. Adv. 2019, 5, eaav7199. 30949579 10.1126/sciadv.aav7199 PMC6447385

[40] M. Maresca , V. G. Lin , N. Guo , Y. Yang , Genome Res. 2013, 23, 539–546. 23152450 10.1101/gr.145441.112 PMC3589542

[41] B. M. Stinson , J. J. Loparo , Annu. Rev. Biochem. 2021, 90, 137–164. 33556282 10.1146/annurev-biochem-080320-110356 PMC8899865

[42] A. Zutz , L. Hamborg , L. E. Pedersen , M. M. Kassem , E. Papaleo , A. Koza , M. J. Herrgård , S. I. Jensen , K. Teilum , K. Lindorff‐Larsen , A. T. Nielsen , Nat. Commun. 2021, 12, 6093. 34667164 10.1038/s41467-021-26337-1 PMC8526717

[43] L. D. Moore , T. Le , G. Fan , Neuropsychopharmacology 2013, 38, 23–38. 22781841 10.1038/npp.2012.112 PMC3521964

[44] W. Wang , X. Zhao , Y. Shao , X. Duan , Y. Wang , J. Li , J. Li , D. Li , X. Li , J. Wong , Sci. Adv. 2021, 7, eabe8511. 34516921 10.1126/sciadv.abe8511 PMC8442919

[45] C. J. Klein , M. V. Botuyan , Y. Wu , C. J. Ward , G. A. Nicholson , S. Hammans , K. Hojo , H. Yamanishi , A. R. Karpf , D. C. Wallace , M. Simon , C. Lander , L. A. Boardman , J. M. Cunningham , G. E. Smith , W. J. Litchy , B. Boes , E. J. Atkinson , S. Middha , B. D. PJ , J. E. Parisi , G. Mer , D. I. Smith , P. J. Dyck , Nat. Genet. 2011, 43, 595–600. 21532572 10.1038/ng.830 PMC3102765

[46] G. Zhong , H. Wang , Y. Li , M. H. Tran , M. Farzan , Nat. Chem. Biol. 2017, 13, 839–841. 28628097 10.1038/nchembio.2410 PMC5577360

[47] H. Wang , B. Rao , J. Lou , J. Li , Z. Liu , A. Li , G. Cui , Z. Ren , Z. Yu , Front. Cell Dev. Biol. 2020, 8, 55. 32117981 10.3389/fcell.2020.00055 PMC7018668

[48] J. Yu , G. G. Chen , P. B. S. Lai , Med. Res. Rev. 2021, 41, 507–524. 33026703 10.1002/med.21738

[49] H. K. Lee , H. M. Lim , S.‐H. Park , M. J. Nam , J. Pers. Med. 2021, 11, 983. 34683124 10.3390/jpm11100983 PMC8540514

[50] W. Sun , J. Wang , Q. Hu , X. Zhou , A. Khademhosseini , Z. Gu , Sci. Adv. 2020, 6, eaba2983. 32490205 10.1126/sciadv.aba2983 PMC7239642

[51] A. Fischer , A. Ehrlich , Y. Plotkin , Y. Ouyang , K. Asulin , I. Konstantinos , C. Fan , Y. Nahmias , I. Willner , Angew. Chem. Int. Ed. 2023, 62, e202311590; 10.1002/anie.202311590 37675854

[52] P. Zhang , A. Fischer , Y. Ouyang , J. Wang , Y. S. Sohn , R. Nechushtai , E. Pikarsky , C. Fan , I. Willner , Chem. Sci. 2021, 12, 14473–14483. 34880998 10.1039/d1sc04229g PMC8580039

