# Supplementary material for: Light‐Triggered CRISPR/Cas12a for Genomic Editing and Tumor Regression
Source: Angew Chem Int Ed Engl. 2025 May 19;64(28):e202502892. doi: 10.1002/anie.202502892 (PMC12232898; doi:10.1002/anie.202502892)
Supplement: Supplementary file 1 — Supporting Information [file ANIE-64-e202502892-s001.pdf]

Supporting Information
©Wiley-VCH 2024
69451 Weinheim, Germany

Light-Triggered CRISPR/Cas12a for Genomic Editing and Tumor Regression

Hong Liu,+[a] Jiantong Dong,+[b] Renzhi Wu,+[a] Jun Dai,*[c] Xiaoding Lou,[a] Fan Xia,[a] Itamar Willner,*[b] and Fujian Huang*[a]

[a] State Key Laboratory of Geomicrobiology and Environmental Changes, Faculty of Materials Science and Chemistry, China University of Geosciences, Wuhan 430074, China

E-mail: huangfj@cug.edu.cn;

[b] Institute of Chemistry, and Center for Nanoscience and Nano-technology, The Hebrew University of Jerusalem, Jerusalem 91904, Israel

E-mail: willnea@vms.huji.ac.il

[c] Department of Obstetrics and Gynecology, Tongji Hospital, Tongji Medical College, Huazhong University of Science and Technology, Wuhan 430034, China

E-mail: jundai@tjh.tjmu.edu.cn

[+] These authors contributed equally to this work.

Table of Contents

1. Experimental Procedures S3

2. Tables 1–4 of oligonucleotide sequences S6

3. Figure S1. Electrophoresis validation of stepwise formation of the Cas12a/OFF-crRNA complex and the Cas12a/ON-crRNA complex S8

4. Figure S2. Electrophoretic comparison of different OFF-crRNA$_{Cyan}$-guided Cas12a background cleavage activities in the absence of light S9

5. Figure S3. Electrophoretic comparison of the LAC12aGE-induced editing of the *Cyan* gene substrate using two different OFF-crRNA$_{Cyan}$ S10

6. Figure S4. Optimization of UV illumination duration for efficient photodeprotection of OFF-crRNA S11

7. Figure S5. Gel electrophoresis comparison of *Cyan* gene editing by the Cas12a and OFF-crRNA versus the always-blocked OFF-crRNA S12

8. Figure S6. Effects of Cas12a/crRNA complex concentration on the cleavage efficiency of *Cyan* gene substrate S13

9. Figure S7. Electrophoretic characterization of the LAC12aGE-induced in vitro editing of *sfGFP* gene in the absence or presence of light S14

10. Figure S8. *sfGFP* gene knockout in HeLa cells transfected with LAC12aGE machinery and the fluorescent dual reporter plasmid S15

11. Figure S9. Workflow for detecting indel frequency by T7E1 assay S16

12. Figure S10. Flow cytometry analysis of transfection efficiency in HEK293T cells S17

13. Figure S11. Effects of different time intervals of UV irradiation on the cell viability of HEK293T cells transfected with LAC12aGE machinery S18

14. Figure S12. Full-length original gel electrophoresis images corresponding to indel frequencies in Figure 3b. S19

15. Figure S13. Full-length original gel electrophoresis images corresponding to indel frequencies in Figure 3c S20

16. Figure S14. Full-length original gel electrophoresis images corresponding to indel frequencies in Figure 3d S21

17. Figure S15. Indel frequencies of LAC12aGE-mediated genome editing activities toward the *VEGFA* gene in HEK293T cells. S22

18. Figure S16. Full-length original gel electrophoresis images corresponding to indel frequencies in Figure 4b S23

19. Figure S17. LAC12aGE-mediated genome editing targeting *HGF* gene in Hep3B cells in the absence and presence of UV illumination. S24

20. Figure S18. Cell proliferation ability of Hep3B cells in different groups measured by EdU assay S25

21. Figure S19. Wound healing assay showing the migration capacities of Hep3B cells in different groups S26

22. Figure S20. Apoptosis analysis of Hep3B cells in different groups using Annexin V-FITC staining assay S27

23. Figure S21. Flow cytometry analysis of apoptosis induced by *HGF* gene knockout in HepG2 and Hep3B cells S28

24. Figure S22. The relative expression levels of *HGF* gene evaluated by RT-qPCR S29

25. Figure S23. Probing the permeability of UV light through mouse skin S30

26. Figure S24. Probing the light-induced deprotection of the OFF-crRNA in the HepG2 tumor-bearing mice S31

27. Figure S25. Indel frequencies resulting from LAC12aGE-mediated genome editing of the *HGF* gene in mice. S32

28. Figure S26. In vivo transfection efficiency assessed by flow cytometry analysis S33

29. Figure S27. Quantitative fluorescence intensities corresponding to images of tumor tissues from different groups of mice in Figure 6f–h S34

30. Figure S28. Histology analysis of major organs excised from HepG2 tumor-bearing nude mice. S35

31. References S36

Experimental Procedures

1. Materials.

Fetal bovine serum (FBS), trypsin, Dulbecco's Modified Eagle's Medium (DMEM), RPMI 1640, MEM/NEAA, PBS, Opti-MEM and Lipofectamine 3000 were purchased from Thermo Fisher Scientific (Waltham, MA). Q5 Hot Start High-Fidelity 2× Master Mix, Nuclease-free Water, T7 Endonuclease I were purchased from New England Biolabs (Beijing, China). QuickExtract DNA Extraction Solution was purchased from Lucigen (USA). Premix Taq were purchased from Takara Biomedical Technology (Beijing, China). Agarose was purchased from Sangon Biotech (Shanghai, China). Hoechst 33342, Gel Red, BeyoClick EdU-555 Cell Proliferation Assay Kit, Mitochondrial Membrane Potential and Apoptosis Detection Kit were purchased from Beyotime Biotechnology (Shanghai, China). TRIpure Reagent was purchased from Aidlab Biotechnologies (Beijing, China). EntiLink 1st Strand cDNA Synthesis Super Mix were purchased from ELK Biotechnology (Wuhan, China). FITC Annexin V Apoptosis Detection Kit were purchased from BD Biosciences (USA). Oligonucleotides were purchased from Hippo Biotechnology Co., Ltd. (Beijing, China).

2. Nucleic acid design and preparation.

The design of the OFF-crRNA was performed by hand and verified by NUPACK. All the nucleic acid strands were dissolved in DEPC water (DNase/RNase free). OFF-crRNA was prepared by a fast-cooling process from 95°C to 4°C in an annealing buffer (20 mM Tris-HCl, 5 mM $MgCl_2$, 150 mM NaCl, pH = 7.5).

3. In vitro cleavage assay.

The cleavage substrate for Cas12a protein was amplified by polymerase chain reaction (PCR) using Premix Taq. 120 nM Cas12a protein was incubated with 120 nM crRNA in 1 × NEBuffer 2.1 for 10 minutes, followed by added 3.0 μL of PCR amplicons per 20.0 μL of reaction system. LAC12aGE assays were performed as above, with the following modifications: 120 nM Cas12a was incubated with 120 nM OFF-crRNA in 1× NEBuffer 2.1 for 10 min, the mixture solutions were irradiated with UV light (λ = 365 nm, 25 mW/cm^2) for 5 minutes; then, added 3.0 μL of PCR amplicons per 20.0 μL of reaction system. Reactions were run on agarose gel electrophoresis and then scanned under UV light using Tanon imaging system (Tanon 5200 Multi). To analyze the efficiency of photodeprotection under varying illumination durations, 120 nM Cas12a was incubated with 120 nM FQ/OFF-crRNA in 1× NEBuffer 2.1. The mixtures were irradiated with UV light for different time intervals, after which the fluorescence intensity was measured using an FS5 fluorescence spectrophotometer (Edinburgh Instruments).

4. Cell culture.

HEK293T, HeLa, U87MG cells were cultured in DMEM medium with 10% FBS and 1% penicillin-streptomycin. HepG2 and Hep3B cells were cultured in MEM/NEAA medium with 10% FBS and 1% penicillin-streptomycin. H460 cells were cultured in RPMI 1640 medium supplemented with 10% FBS and 1% penicillin-streptomycin at 37 °C with 5% CO_2.

5. LAC12aGE-mediated editing of an exogenous dual fluorescent reporter gene.

HEK293T and HeLa cells were plated on confocal dishes and cultured to 70 ~ 90% confluence, and the culture medium was replaced with fresh culture medium prior to transfection. 0.5 μg of AsCas12a-encoding plasmid and 0.5 μg of sfGFP/mCherry dual fluorescent reporter plasmid were transfected into cells with Lipofectamine 3000 in Opti-MEM I reduced serum medium following the manufacturer's recommended protocol. After 12 hours, 50.0 pmol OFF-crRNA was transfected into cells by Lipofectamine 3000. After 6 hours, the cells were irradiated for 5 minutes with UV light (λ = 365 nm, 25 mW/cm^2). Cells were incubated for 72 hours at 37°C with 5% CO_2. Cells were washed three times with PBS and imaged on a laser scanning confocal microscope (Zeiss LSM 880). For the spatial control experiments, UV irradiations were performed through a photo mask to only expose a portion of cells to 365 nm light for 5 minutes, and then the cells in the whole well were cultured for another 72 hours and imaged with a confocal microscope. The fluorescence intensity of each cell was quantified using ImageJ software (mean gray intensity = the whole intensity of the cell/the area of the cell).

6. LAC12aGE-mediated endogenous gene editing.

The cells were plated on 24-well plates and cultured to 70–90% confluence, and the culture medium was replaced with fresh culture medium prior to transfection. 0.5 μg of AsCas12a-encoding plasmid was transfected into cells with Lipofectamine 3000 in Opti-MEM I reduced serum medium following the manufacturer's recommended protocol. After 12 hours, 50.0 pmol OFF-crRNA was transfected into cells by Lipofectamine 3000. After 6 hours, the cells were irradiated for 5 minutes with UV light (λ = 365 nm, 25 mW/cm^2). After the cells were cultured at 37°C and 5% CO_2 incubator for another 72 hours, the cells were collected for genomic DNA isolation and analyzed by T7 endonuclease I (T7E1) assays to assess the LAC12aGE-mediated Indel frequency.

7. Indel analysis by T7E1 assay.

Genomic DNA was extracted using the QuickExtract DNA Extraction Solution following the manufacturer's protocol. Genomic regions flanking the CRISPR target site for each gene was amplified using gDNA template, Q5 hot start high-fidelity DNA polymerase and specific primers on an Applied Biosystems 2720 thermal cycler (Thermo Fisher). The PCR products were re-annealed in NEBuffer 2 (50 mM NaCl, 10 mM Tris-HCl, 10 mM $MgCl_2$, 1 mM DTT, pH 7.9), heated to 95°C for 10 minutes and then slowly cooled to room temperature. Subsequently, the annealed samples were digested by T7 Endonuclease I at 37 °C for 30 min and analyzed by agarose gel electrophoresis. Gels were stained with Gel Red and imaged with Tanon imaging system (Tanon 5200 Multi). The cutting products were analyzed to calculate indel efficiency using the Image J software. Indel percentage was determined by the formula[1]

(% Indel = 100 × [1-(1-fraction cleaved)$^{1/2}$]), where fraction cleaved is equal to the concentration of cleaved DNA divided by the total concentration of DNA.

8. Cell migration, cell proliferation and cell apoptosis assays.

For cell migration assay, HepG2 and Hep3B cells were seeded at 6-well plates, and after LAC12aGE assay treatment, the center of the wells was scratched with a pipette tip, and then the cells were rinsed with PBS to remove cell debris. The wound area closure was observed using a fluorescence microscope (Olympus). For cell proliferation assay, HepG2 cells and Hep3B cells were seeded on confocal dishes and subjected to LAC12aGE assay. Subsequently, BeyoClick EdU-555 Cell Proliferation Assay Kit was used to detect cell proliferation visually according to the manufacturer's instruction. To assess the induction of the apoptotic cells after the treatment with the LAC12aGE assay, the cells were incubated with FITC Annexin V Apoptosis Detection Kit according to the manufacturer's protocol. The stained cells were immediately acquired using a Agilent NovoCyte Flow Cytometer (Agilent) and were analyzed by NovoExpress. To investigate the apoptotic cells using microscopy, the cells were seeded on confocal dishes and subjected to LAC12aGE assay. Subsequently, Mitochondrial Membrane Potential and Apoptosis Detection Kit with Mito-Tracker Red CMXRos and Annexin V-FITC was used to analyze cell apoptosis according to the manufacturer's instruction.

9. Cell viability test.

The standard MTT assays was operated to evaluate the cytotoxicity of LAC12aGE. HEK293T cells were seeded in a 96-well plate (1×10^4 cells per well) and grown overnight. The cells were subjected to LAC12aGE with various irradiation time (0, 1, 3, 5, 10, 15 or 20 min) and then cultured for another 72 h. After that, a total 10 μL of MTT solution (5 mg/ml) was added into every well incubated at 37 °C for 4 h. Finally, the supernatants were aspirated and then 150 μL of DMSO was added to each well. The absorbance values of the wells at 490 nm were performed on a microplate reader.

10. RT-qPCR analysis

Total RNA of cells was extracted using the TRIpure Reagent according to the manufacturer's instructions. The cDNA was prepared by using EntiLink 1st Strand cDNA Synthesis Super Mix according to the indicated protocol. qPCR analysis was performed using the EnTurbo SYBR Green PCR Super Mix. PCR amplification was implemented on the Applied Biosystems QuantStudio 6 Flex (Thermo Fisher Scientific). Program for qPCR amplifications were as follows: 95°C for 30 s, followed by 40 cycles at 95°C for 10 s, 58°C for 30 s, and 72°C for 30 s. Samples were normalized to the housekeeping gene glyceraldehyde 3-phosphate dehydrogenase (GAPDH) as the endogenous control. Standard ΔΔCt method was used to obtain relative expression of cell samples.

11. Gene editing mediated by the LAC12aGE system in tumor mouse model.

Female BALB/c nude mice (5～6 weeks old) were purchased from Beijing Vital River Laboratory Animal Technology Co., Ltd. This study was approved by the Committee on Ethics of Animal Experiments of Tongji Hospital, Tongji Medical College, Huazhong University of Science and Technology (Ethics No. TJH-202106016). To establish a subcutaneous tumor model, 1×10^7 HepG2 cells were subcutaneously implanted into the nude mice for further use. The sizes of subcutaneous tumors were measured and the volume was calculated following the formula: volume = (length × width^2) / 2. When the tumor size reached about 80 to 100 mm^3, the tumor-bearing mice were randomly divided into six groups: PBS, PBS + UV, Cas12a/OFF-crRNA$_{Cyan}$, Cas12a/OFF-crRNA$_{Cyan}$ + UV, Cas12a/OFF-crRNA$_{HGF}$, Cas12a/OFF-crRNA$_{HGF}$ + UV. Mice were injected intratumorally with 50 μL Cas12a plasmid (10 μg) for 12 h and then injected intratumorally with OFF-crRNA (15 μg) for 6 h. In vivo-jetPEI (Polyplus) was used for injection of mouse tumors following manufacturer's recommendations. After the injection, the tumor was irradiated by a 365 nm light (λ = 40 mW/cm^2, 10 min). The treatments were implemented every 3 days. The administration was monitored for up to 21 days, and during that time, the body weights and tumor volumes were recorded for further analysis, and then animals were euthanized on day 21. Single-cell suspension preparation was performed as follows: the excised tumor tissue was finely minced using surgical scissors and resuspended in a digestion solution containing 0.08% trypsin and 0.1% collagenase II. The tissue suspension was incubated at 37°C for 30 min, followed by filtration through a 200-mesh strainer to obtain a single-cell suspension. To analyze photodeprotection occurring within the tumor, tumor-bearing nude mice were injected with Cas12a plasmid and FQ/OFF-crRNA intratumorally. Bioluminescence images of the mice were subsequently acquired using the IVIS Spectrum imaging system (PerkinElmer).

12. Flow Cytometry Analysis

The Cas12a-EGFP fusion plasmid was transfected into HEK293T cells or administered intratumorally in Xenograft BALB/c nude mice bearing HepG2 tumors. After 72 hours of treatment, HEK293T cells were harvested or single-cell suspensions were prepared from collected tumor tissues, followed by immediate analysis using an Agilent NovoCyte Flow Cytometer (Agilent). Data were analyzed using NovoExpress software.

13. H&E and TUNEL staining.

For H&E staining, subcutaneous tumors and major organs that stem from mice after different treatments were harvested. After fixing in 4% paraformaldehyde, the collected tumors and organs were embedded in paraffin, sectioned into about 4 μm, and lastly stained with hematoxylin-eosin (H&E). Moreover, TUNEL BrightGreen Apoptosis Detection Kit (Vazyme) was applied to determine tumor cell apoptosis on tumor slices. DAPI was used to stain the nuclei of the cells. The samples were observed via fluorescence microscope.

14. Ki67 and HGF staining.

The obtained tumor tissues were cut into 4 μm-thick sections for immunofluorescence staining. After deparaffinization, antigen retrieval, and BSA blocking, the tissue sections were incubated with Ki67 primary antibodies (Cell Signaling Technology) and HGF primary antibody (ABclonal Biotechnology) at 4°C overnight, followed by washing 3 times with TBST. Then the Alexa Fluor 488 donkey anti-rabbit IgG (Thermo Fisher Scientific) was added dropwise, and incubation was continued for 45 min at 37°C. The tissue sections were washed with TBST for 3 times again. DAPI was used to stain the nuclei of the cells. The sections were observed via fluorescence microscope.

15. Statistical analysis.

Results were presented as means ± SD. Comparisons between the groups were performed using a Student's *t* test. Differences were considered statistically significant at $P < 0.05$ (*), very significant at $P < 0.01$ (**), and extremely significant at $P < 0.001$ (***). The OriginPro 9.1 software was used for statistical analysis. *n* and *P* values are provided in the figure legends.

2. Supporting tables

Table S1. Sequences of target gene substrates in this study.

Name	Sequence (5’-3’)
Cyan gene substrate	TACGAAGGGACGCAGACCTCGACTTTTAAAGTCACCATGGCCAACGGTGGGCCCCATGCATTCTCCTTTGACATACTATCTACAGT<br>GTTCATGTATGGAAATCGATGCTTTACTGCGTATCCTACCAGTATGCCCGACTATTTCAAACAAGCATTTCCTGACGGAATGTCATA<br>TGAAAGGACTTTTACCTATGAAGATGGAGGAGTTGCTACAGCCAGTTGGGAAATAAGCCTTAAAGGCAACTGCTTTGAGCACAAAT<br>CCACGTTTCATGGAGTGAACTTTCCTGCTGATGGACCTGTGATGGCGAAGATGACAACTGGTTGGGACCCATCTTTTGAGAAAATG<br>ACTGTCTGCGATGGAATATTGAAGGGTGATGTCACCGCGTTCCTCATGCTGCAAGGAGGTGGCAATTACAGATGCCAATTCCACA<br>CTTCTTACAAGACAAAAAAACCGGTGACGATGCCACCAAACCATGCGGTGGAACATCGCATTGCGAGGACCGACCTTGACAAAGG<br>TGGCAACAGTGTTCAGCTGACGGAGCACGCTGTTGCACATATAACCTCTGTTGTCCCTTTCGC
sfGFP gene substrate	CTCAACCCTATCTCGGTCTATTCTTTTGATTTATAAGGGATTTTGCCGATTTCGGCCTATTGGTTAAAAAATGAGCTGATTTAACAAA<br>AATTTAACGCGAATTTTAACAAAATATTAACGCTTACAATTTACGCGTTAAGATACATTGATGAGTTTGGACAAACCACAACTAGAAT<br>GCAGTGAAAAAAATGCTTTATTTGTGAAATTTGTGATGCTATTGCTTTATTTGTAACCATTATAAGCTGCAATAAACAAGTTAACAAC<br>AACAATTGCATTCATTTTATGTTTCAGGTTCAGGGGGAGGTGTGGGAGGTTTTTTAAAGCAAGTAAAACCTCTACAAATGTGGTATG<br>GCTGATTATGATCAGTTATCTAGATCCGGTGGATCCTTACTTGTACAGCTCGTCCATGCCGTGAGTGATCCCGGCGGCGGTCACG<br>AACTCCAGCAGGACCATGTGATCGCGCTTCTCGTTGGGGTCTTTGCTCAGCACGGACTGGGTGCTCAGGTAGTGGTTGTCGGGC<br>AGCAGCACGGGGCCGTCGCCGATGGGGGTGTTCTGCTGGTAGTGGTCGGCGAGCTGCACGCTGCCGTCCTCCACGTTGTGGCG<br>GATCTTGAAGTTGGCCTTGATGCCGTTCTTCTGCTTGTCGGCGGTGATATAGACGTTGTGGCTGTTGAAGTTGTACTCCAGCTTGT<br>GCCCCAGGATGTTGCCGTCCTCCTTGAAGTCGATGCCCTTCAGCTCGATGCGGTTCACCAGGGTGTCGCCCTCGAACTTCACCTC<br>GGCGCGGGTCTTGTAGGTGCCGTCGTCCTTGAAGCTGATGGTGCGCTCCTGGACGTAGCCTTCGGGCATGGCGGACTTGAAGAA<br>GTCGTGGCGCTTCATGTGGTCGGGGTAGCGGCTGAAGCACTGCACGCCGTAGGTCAGGGTGGTCACGAGGGTGGGCCAGGGCA<br>CGGGCAGCTTGCCGGTGGTGCAGATGAACTTCAGGGTCAGCTTGCCGTTGGTGGCATCGCCCTCGCCCTCGCCGCGCACGCTG<br>AACTTGTGGCCGTTTACGTCGCCGTCCAGCTCGACCAGGATGGGCACCACCCCGGTGAACAGCTCCTCGCCCTTGCTCACCATAA<br>GCTTGCTTCCGCCTCCTCCACTACCTCCACCTCCAGCTCGAGATCTGAGTCCGGACTTGTACAGCTCGTCCATGCCGCCGGTGGA<br>GTGGCGGCCCTCGGCGCGTTCGTACTGTTCCACGATGGTGTAGTCCTCGTTGTGGGAGGTGATGTCCAACTTGATGTTGACGTTG<br>TAGGCGCCGGGCAGCTGCACGGGCTTCTTGGCCTTGTAGGTGGTCTTGACCTCAGCGTCGTAGTGGCCGCCGTCCTTCAGCTTC<br>AGCCTCTGCTTGATCTCGCCCTTCAGGGCGCCGTCCTCGGGGTACATCCGCTCGGAGGAGGCCTCCCAGCCCATGGTCTTCTTC<br>TGCATTACGGGGCCGTCGGAGGGGAAGTTGGTGCCGCGCAGCTTCACCTTGTAGATGAACTCGCCGTCCTGCAGGGAGGAGTCC<br>TGGGTCACGGTCACCACGCCGCCGTCCTCGAAGTTCATCACGCGCTCCCACTTGAAGCCCTCGGGGAAGGACAGCTTCAAGTAG<br>TCGGGGATGTCGGCGGGGTGCTTCACGTAGGCCTTGGAGCCGTACATGAACTGAGGGGACAGGATGTCCCAGGCGAAGGGCAG<br>GGGGCCACCCTTGGTCACCTTCAGCTTGGCGGTCTGGGTGCCCTCGTAGGGGCGGCCCTCGCCCTCGCCCTCGATCTCGAACTC<br>GTGGCCGTTCACGGAGCCCTCCATGTGCACCTTGAAGCGCATGAACTCCTTGATGATGGCCATGTTATCCTCCTCGCCCTTGCTC<br>ACCATGGTGGCGACCGGTAGCGCTAGCGGATCTGACGGTTCACTAAACCAGCTCTGCTTATATAGACCTCCCACCGTACACG
DNMT1 gene substrate	CGTTTCCCTCACTCCTGCTCGGTGAATTTGGCTCAGCAGGCACCTGCCTCAGCTGCTCACTTGAGCCTCTGGGTCTAGAACCCTC<br>TGGGGACCGTTTGAGGAGTGTTCAGTCTCCGTGAACGTTCCCTTAGCACTCTGCCACTTATTGGGTCAGCTGTTAACATCAGTACG<br>TTAATGTTTCCTGATGGTCCATGTCTGTTACTCGCCTGTCAAGTGGCGTGACACCGGGCGTGTTCCCCAGAGTGACTTTTCCTTTT<br>ATTTCCCTTCAGCTAAAATAAAGGAGGAGGAAGCTGCTAAGGACTAGTTCTGCCCTCCCGTCACCCCTGTTTCTGGCACCAGGAAT<br>CCCCAACATGCACTGATGTTGTGTTTTTAACATGTCAATCTGTCCGTTCACATGTGTGGTACATGGTGTTTGTGGCCTTGGCTGACA<br>TGAAGCTGTTGTGTGAGGTTCGCTTATCAACTAATGATTTAGTGATCAAATTGTGCAGTACTTTGTGCATTCTGGATTTTAAAAGTTT<br>TTTATTATGCATTATATCAAATCTACCACTGTATGAGTGGAAATTAAGACTTTATGTAGTTTTTATATGTTGTAATATTTCTTCAAATAA<br>ATCTCTCCTATAAACCACCCTGGGTGGGTATTCGTGATTTGCACGGGACTTC
VEGFA gene substrate	CTCTGCGGACGCCCAGTGAAGCCTGGCCCGCAGCCCGCCGCAATGAAGGGGAAGCTCGACCCCCACCAAGGTTCACAGCCTGA<br>AAATTACCCATCCGCCCCCGGAAACTCTGTCCAGAGACACGCGCCCGCGGGGCATTGGCGAGGAGGGAGCAGGAAAGTGAGGT<br>TACGTGCGGACAGGGCCTGAGAGCCGTTCCCTCTTTGCTAGGAATATTGAAGGGGGCAGGGGAAGGCGGAGAGCCGGACAGGG<br>ACGGGTGGGGAGAGGGACACACAGATCTATTGGAATCCTGGAGTGACCCCTGGCCTTCTCCCCGCTCCAACACCCTCAACCCCA<br>CACGCACACACTCACTCACCCACACAGACACACACGTCCTCACTCTCGAAGACGCTGCTCGCTCCATTCACCCAGCTTCCCTGTG<br>GTGGCCGAGCGCCCCCTAGTGACTGCCGTCTGCACACCCCGGCTCTGGCTAAAGAGGGAATGGGCTTTGGAAAGGGGGTGGGG<br>GGAGTTTGCTCCTGGACCCCCTATTTCTGACCTCCCAAACAGCTACA
HGF gene substrate	TTCCATAACCAAAGCCACTGTGGATTTTAGAAGGTAGCAGTTTTTGCGCTCTCCCTTACTCAAGCTATGTTTCCATAAACTGAGAGC<br>TAAGTCTTCGTTTGTAGTGAGTTAAATCTAGAGTCAAACTTACAATTTTATGATCAAATCACATTGACTACAACACAAAAGACACATG<br>ATTATAATACATGCATGCTATATTTGGAAAATTATTCTAAGAAGAAAATGAAGTAACTTACTTGCAAGTGAATGGAAGTCCTTTATTC<br>CTAGTACATCTATTAGCACATTGGTCTGCAGTATTCACTTTTTTGGTTTTTATCTTCAGTGCTGGATCTATTTTGATTAGGGTAGTCTT<br>TGCTGATTTTTTGAATTCATGAATTGTATTTCTTCTTTTCCTTTGTCCCTCTATTAAATACAAAATGTTTTAAAAAAATAAACATTGGAG<br>AAATGTTTTTAAGGCTCATATATAAAAAAAATCCTAAGGATCATCTACAAGAAAGTGATTTTACTTGACAATATACTAGATTTCCAGG<br>GGTTAGGAGCAGAGTGA

Table S2. Sequences of crRNAs and caged OFF-crRNAs used for constructing the LAC12aGE systems.

Name	Sequence (5'-3')
crRNA$_{sfGFP}$	UAAUUUCUACUCUUGUAGAUCGUCGCCGUCCAGCUCGACCAGG
crRNA$_{DNMT1}$	UAAUUUCUACUCUUGUAGAUCUGAUGGUCCAUGUCUGUUACUC
crRNA$_{VEGFA}$	UAAUUUCUACUCUUGUAGAUCUAGGAAUAUUGAAGGGGGCAGG
crRNA$_{HGF}$	UAAUUUCUACUCUUGUAGAUUCUUCAGUGCUGGAUCUAUUUUG
OFF-crRNA$_{Cyan1}$	AAUAACACUU/**PC**/AGUAGA/**PC**/UAAUUUCUACUAAGUGUAGAUAUGGAGUGAACUUUCCUGCUGAU
OFF-crRNA$_{Cyan2}$	CCCAUCUACACU/**PC**/AGUAGAAAGUAAUUUCUACUAAGUGUAGAUAUGGAGUGAACUUUCCUGCUGAU
OFF-crRNA$_{Cyan3}$	AAUAACACUUAGUAGA/**PC**/UAAUUUCUACUAAGUGUAGAUAUGGAGUGAACUUUCCUGCUGAU
OFF-crRNA$_{sfGFP}$	AAUAUACAAGAGUAGA/**PC**/UAAUUUCUACUCUUGUAGAUCGUCGCCGUCCAGCUCGACCAGG
OFF-crRNA$_{DNMT1}$	AAUAUACAAGAGUAGA/**PC**/UAAUUUCUACUCUUGUAGAUCUGAUGGUCCAUGUCUGUUACUC
OFF-crRNA$_{VEGFA}$	AAUAUACAAGAGUAGA/**PC**/UAAUUUCUACUCUUGUAGAUCUAGGAAUAUUGAAGGGGGCAGG
OFF-crRNA$_{HGF}$	AAUAUACAAGAGUAGA/**PC**/UAAUUUCUACUCUUGUAGAUUCUUCAGUGCUGGAUCUAUUUUG
FQ/OFF-crRNA	AAUAUACAAGAGUA/**PC**/GA/**dTBHQ2**//**PC**/U/**Cy5**/AAUUUCUACUCUUGUAGAUUCUUCAGUGCUGGAUCUAUUUUG

Table S3. Primer sequences used for PCR amplification of target gene substrates for gel electrophoresis and T7E1 experiments.

Name	Sequence (5'-3')
Cyan-F	TACGAAGGGACGCAGACC
Cyan-R	GCGAAAGGGACAACAGAGG
sfGFP-F	CTCAACCCTATCTCGGTCTATTCTT
sfGFP-R	CGTGTACGGTGGGAGGTCTAT
DNMT1-F	CGTTTCCCTCACTCCTGC
DNMT1-R	GAAGTCCCGTGCAAATCAC
VEGFA-F	CTCTGCGGACGCCCAGTGAA
VEGFA-R	TGTAGCTGTTTGGGAGGTCAGAAAT
HGF-F	TTCCATAACCAAAGCCACT
HGF-R	TCACTCTGCTCCTAACCC

Table S4. Primer sequences used for RT-qPCR analysis to measure the gene expression in cells.

Name	Sequence (5'-3')
GAPDH-F	CATCATCCCTGCCTCTACTGG
GAPDH-R	GTGGGTGTCGCTGTTGAAGTC
HGF-F	GGGCAACTTATCCCAAACAAG
HGF-R	GCACAAGATATTACGGGATGGT

3. Supporting figures

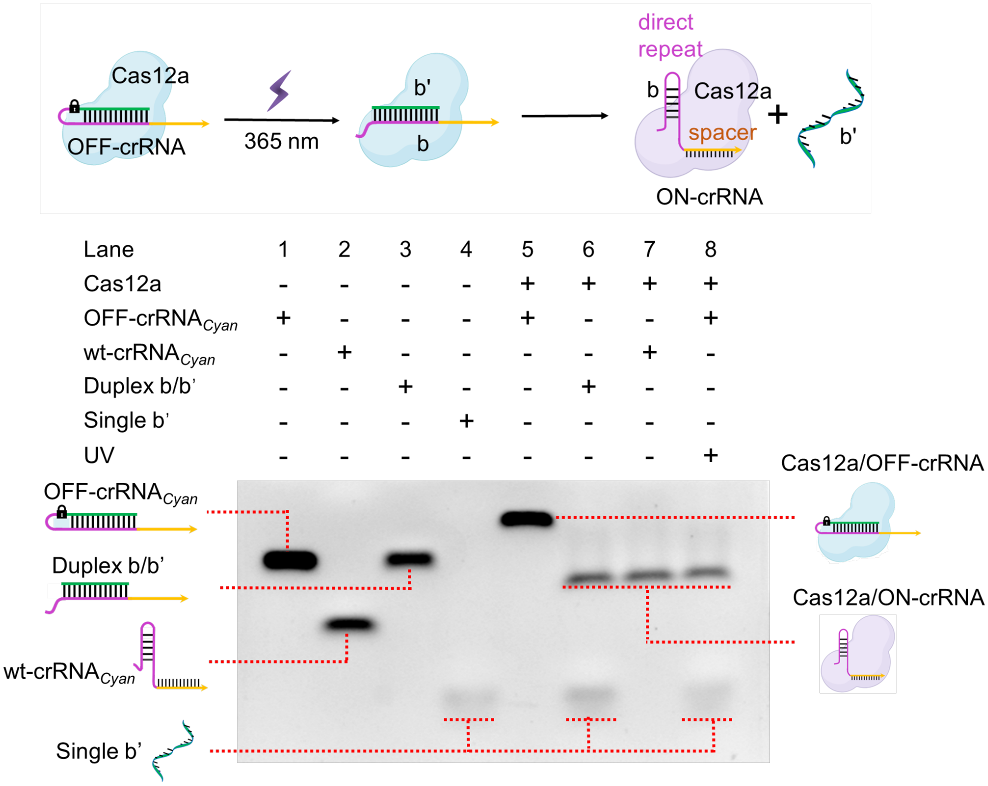

Figure S1. Gel electrophoresis validation of stepwise formation of the Cas12a/OFF-crRNA complex and its phototriggered activation into the Cas12a/ON-crRNA complex. Lane 1, OFF-crRNA$_{Cyan}$ (1 µM); Lane 2, wt-crRNA$_{Cyan}$ (1 µM); Lane 3, duplex b/b' (1 µM); Lane 4, single b' (1 µM); Lane 5, Cas12a/OFF-crRNA$_{Cyan}$ (1.5 µM) without UV illumination; Lane 6, Cas12a/duplex b/b' (1.5 µM); Lane 7, Cas12a/wt-crRNA$_{Cyan}$ (1.5 µM); Lane 8, Cas12a/OFF-crRNA$_{Cyan}$ (1.5 µM) with UV illumination.

Figure S1 presents the stepwise gel electrophoresis results confirming the formation of the inactive Cas12a/OFF-crRNA complex and its light-induced transition into the active Cas12a/ON-crRNA complex. Lanes 1–4 present bands corresponding to the control constituents (photocaged hairpin OFF-crRNA$_{cyan}$, wt-crRNA$_{cyan}$, photocleaved b/b' duplex, and separated single strand b') in the absence of Cas12a. The wt-crRNA$_{cyan}$ migrates faster than the caged OFF-crRNA$_{cyan}$ and photocleaved duplex b/b', consistent with its lower molecular weight. Lane 5 presents the band of the non-illuminated Cas12a/OFF-crRNA$_{cyan}$ complex. Evidently, the migration of this complex is hindered compared to the free caged hairpin, due to its higher molecular weights, confirming that the caged OFF-crRNA$_{cyan}$ hairpin is indeed associated with Cas12a prior to illumination. Lanes 6 and 7 present control bands corresponding to the association of the duplex b/b' to Cas12a (lane 6), and the single wt-crRNA$_{cyan}$ bound to Cas12a (lane 7). Lane 8 depicts the bands corresponding to the 365 nm-photoactivated transition of Cas12a/OFF-crRNA$_{cyan}$ into Cas12a/ON-crRNA$_{cyan}$. Evidently, the original inactive complex band is depleted, and a faster migrating band appears, indicating the formation of a lower molecular weight product. The migration pattern of the phototriggered Cas12a/ON-crRNA$_{cyan}$ band aligns with the control bands in lanes 6 and 7, confirming that the light-induced activation successfully generated the Cas12a/ON-crRNA$_{cyan}$ complex. Moreover, lane 8 presents a rapidly migrating band, consistent with the non-protein associated strand b', further validating the light-triggered stepwise separation of the duplex b/b', as confirmed by the migration of free b' in lanes 4 and 6.

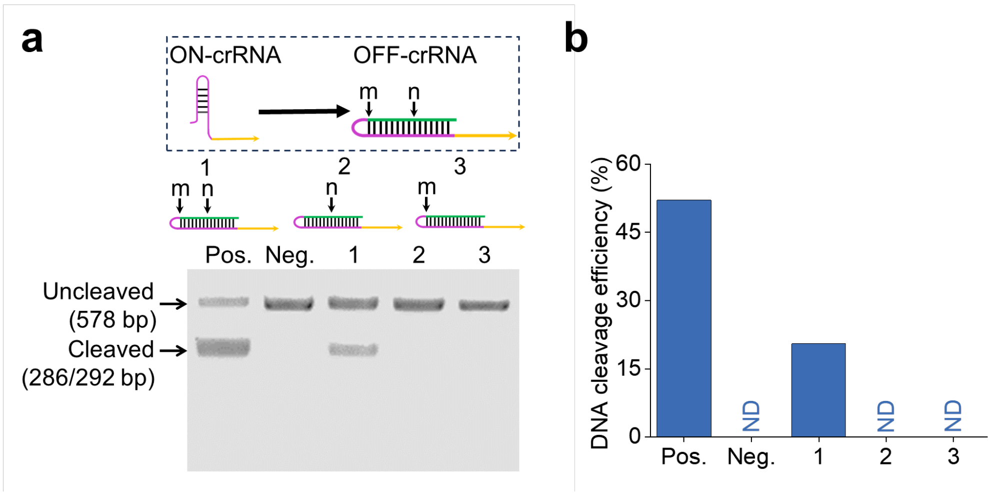

Figure S2. Electrophoretic comparison of different OFF-crRNA$_{Cyan}$-guided Cas12a background cleavage activities in the absence of light. (a) 2.5% Agarose gel electrophoresis characterizing the in vitro gene-editing products. The uncleaved *Cyan* gene substrate is 578 bp, which can be cleaved into two fragments composed of 286 bp and 292 bp. Neg., negative control using Cas12a in the absence of crRNA$_{Cyan}$. Pos., positive control using Cas12a and wt-crRNA$_{Cyan}$. 1, OFF-crRNA$_{Cyan1}$; 2, OFF-crRNA$_{Cyan2}$; 3, OFF-crRNA$_{Cyan3}$. Site m, modification of a photocleavable (PC) linker at the 5' end of the crRNA's direct repeat region; Site n, modification of a PC linker at the extension of the 5' end of the crRNA's direct repeat region. (b) Quantification of in vitro DNA cleavage efficiency from (a) using ImageJ software. The DNA cleavage efficiency (%) was calculated as 100% × $[1-(1-\text{fraction cleaved})^{1/2}]$, where fraction cleaved is equal to the concentration of the cleaved DNA divided by the total concentration of DNA.

In the absence of light (λ = 365 nm) activation, the OFF-crRNA$_{Cyan1}$ produces a strong background cleavage activity, whereas OFF-crRNA$_{Cyan2}$ and OFF-crRNA$_{Cyan3}$ do not guide Cas12a to cleave the *Cyan* gene substrate, demonstrating the leakage-free design of OFF-crRNA$_{Cyan2}$ and OFF-crRNA$_{Cyan3}$.

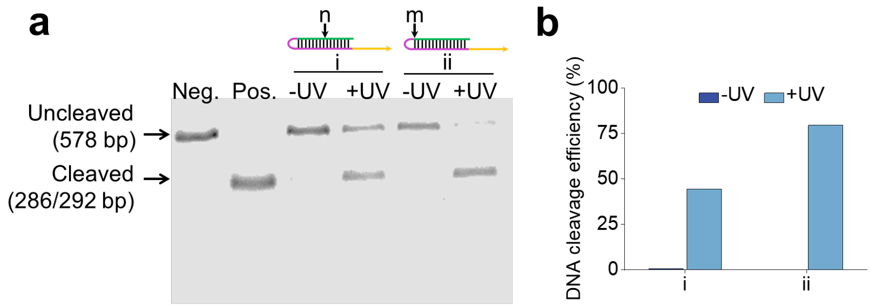

Figure S3. Electrophoretic comparison of the LAC12aGE-induced editing of the *Cyan* gene substrate using two different OFF-crRNA$_{Cyan}$. (a) 2.5% Agarose gel electrophoresis characterizing the in vitro gene-editing products. The uncleaved *Cyan* gene substrate is 578 bp, which can be cleaved into two fragments composed of 286 bp and 292 bp. Neg., negative control using Cas12a in the absence of crRNA$_{Cyan}$. Pos., positive control using Cas12a and wt-crRNA$_{Cyan}$. i, OFF-crRNA$_{Cyan2}$; ii, OFF-crRNA$_{Cyan3}$. (b) Quantification of in vitro DNA cleavage efficiency from (a) using ImageJ software. The DNA cleavage efficiency (%) was calculated as 100% × [1-(1-fraction cleaved)$^{1/2}$], where fraction cleaved is equal to the concentration of the cleaved DNA divided by the total concentration of DNA.

Upon UV irradiation, OFF-crRNA$_{Cyan3}$ guided more efficient cleavage of the *Cyan* gene substrate by Cas12a compared to OFF-crRNA$_{Cyan2}$. Therefore, OFF-crRNA$_{Cyan3}$ was selected as the precursor-yielding crRNA for the light-stimulated activation of the LAC12aGE machinery.

Optimization of UV illumination duration for efficient photodeprotection of OFF-crRNA

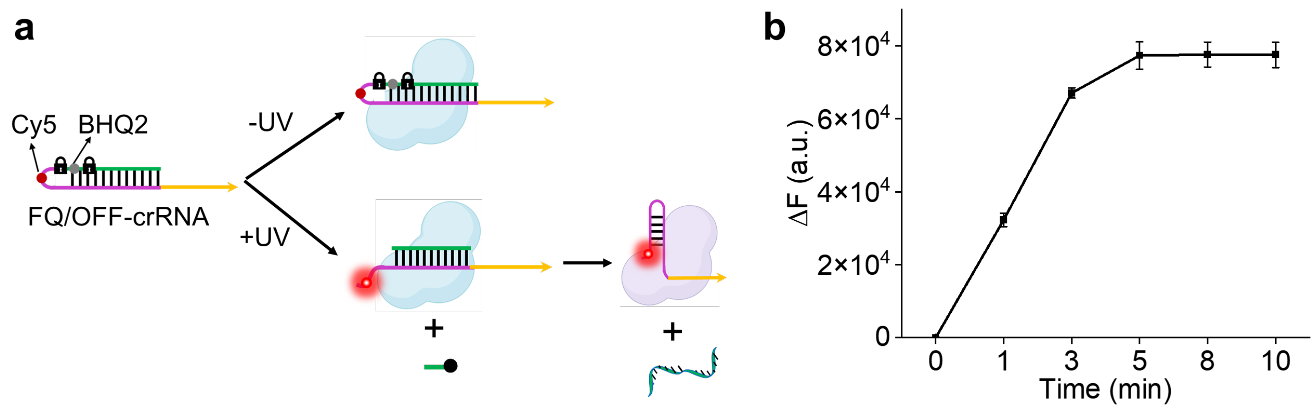

Figure S4. (a) Schematic illustration of the photodeprotection evaluation of the LAC12aGE system using a Cy5/BHQ2-labeled photocaged OFF-crRNA (FQ/OFF-crRNA). (b) Fluorescence enhancement resulting from photodeprotection of FQ/OFF-crRNA under varying durations of UV illumination. Data are presented as mean ± SD (n = 3). (λ = 365 nm, 25 mW/cm^2).

To optimize the UV illumination duration for effective photodeprotection, we employed a Cy5/BHQ2-labeled OFF-crRNA (FQ/OFF-crRNA), photocaged with an *o*-nitrobenzyl phosphate ester, in combination with Cas12a, together constituting the core components of the LAC12aGE system (Figure S4a). Upon UV exposure, the photocage was removed, leading to the separation of the BHQ2 quencher from Cy5 and resulting in fluorescence recovery. As shown in Figure S4b, fluorescence intensity increased with longer irradiation times, reaching saturation at approximately 5 minutes. This indicates that a 5-minute UV exposure is sufficient to achieve complete photodeprotection of FQ/OFF-crRNA.

Lane	1	2	3	4	5	6
Cyan gene substrate	+	+	+	+	+	+
Cas12a	-	+	+	+	+	+
OFF-crRNA$_{Cyan}$	-	+	-	+	-	-
wt-crRNA$_{Cyan}$	-	-	-	-	+	-
Always blocked-crRNA	-	-	+	-	-	+
UV (365 nm)	-	-	-	+	-	+

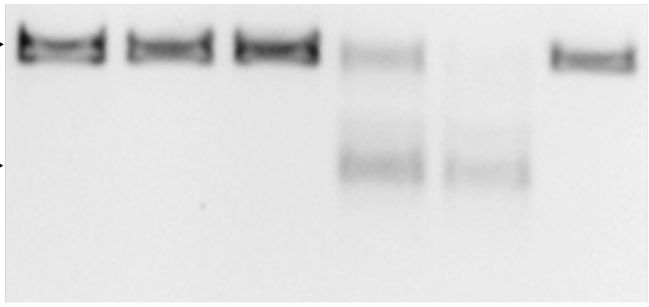

Figure S5. Gel electrophoresis comparison of *Cyan* gene editing by the Cas12a and OFF-crRNA versus the always-blocked OFF-crRNA lacking a photo-responsive site. Lane 1, *Cyan* gene substrate; Lane 2, *Cyan* gene substrate incubated with Cas12a/OFF-crRNA$_{Cyan}$ (120 nM) without UV illumination; Lane 3, *Cyan* gene substrate incubated with Cas12a/always blocked-crRNA$_{Cyan}$ (120 nM) without UV illumination; Lane 4, *Cyan* gene substrate incubated with Cas12a/OFF-crRNA$_{Cyan}$ (120 nM) with UV illumination; Lane 5, *Cyan* gene substrate incubated with Cas12a/wt-crRNA$_{Cyan}$ (120 nM); Lane 6, *Cyan* gene substrate incubated with Cas12a/always blocked-crRNA$_{Cyan}$ (120 nM) with UV illumination. *Cyan* gene substrate is 3.0 μL of PCR amplicons per 20.0 μL of reaction system. Incubation time: 60 min.

Effects of Cas12a/crRNA complex concentration on the in vitro gene cleavage efficiency

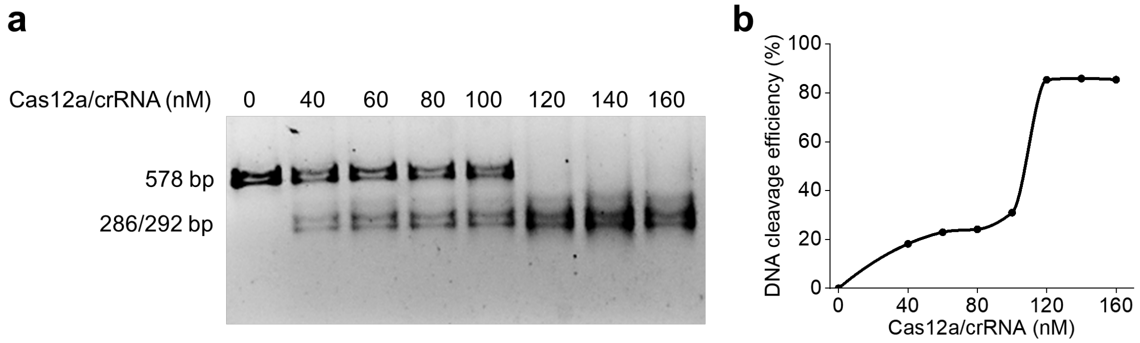

Figure S6. Effects of Cas12a/crRNA complex concentration (Cas12a:crRNA=1:1) on the cleavage efficiency of *Cyan* gene substrate. (a) Gel electrophoresis of cleaved products generated upon subjecting the Cyan fluorescent protein gene to the Cas12a/crRNA$_{cyan}$ complex for 60 min. The uncleaved *Cyan* gene substrate is 578 bp, which can be cleaved into two fragments composed of 286 bp and 292 bp. (b) Cleavage efficiency of the *Cyan* gene by different concentrations of the Cas12a/crRNA$_{cyan}$ complex. The DNA cleavage efficiency (%) was calculated as 100% × [1-(1-fraction cleaved)$^{1/2}$], where fraction cleaved is equal to the concentration of the cleaved DNA divided by the total concentration of DNA.

The efficacy of *Cyan* gene cleavage by the Cas12a/crRNA complex over 60 min was probed using different 1:1 molar concentrations of the Cas12a and crRNA constituents, with a range of 40–160 nM. The results of cleavage were analyzed by gel electrophoresis and are presented in Figure S6. Evidently, at lower concentrations, cleavage remained incomplete within this time frame (60 min), whereas at a concentration of 120 nM and higher, cleavage efficiency reached ca. 85%. Based on these results, a Cas12a/crRNA concentration of 120 nM was selected as the optimal condition for in vitro studies.

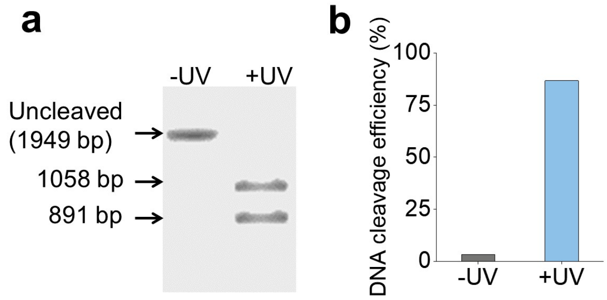

Figure S7. Electrophoretic characterization of LAC12aGE-induced in vitro editing of *sfGFP* gene in the absence or presence of light. (a) 1.5% Agarose gel electrophoresis characterizing the products of in vitro gene editing of *sfGFP* gene. (b) Quantification of in vitro DNA cleavage efficiency from (a) using ImageJ software. The DNA cleavage efficiency (%) was calculated as 100% × [1-(1-fraction cleaved)$^{1/2}$], where fraction cleaved is equal to the concentration of the cleaved DNA divided by the total concentration of DNA.

LAC12aGE machinery-mediated in vitro editing and cleavage of *sfGFP* gene was tested using OFF-crRNA$_{sfGFP}$ as the basic component of LAC12aGE. The results showed that, upon light activation, the LAC2aGE machinery cleaved the *sfGFP* gene (1949 bp) into two fragment products of 1058 bp and 891 bp, with a DNA cleavage efficiency of 86%.

.

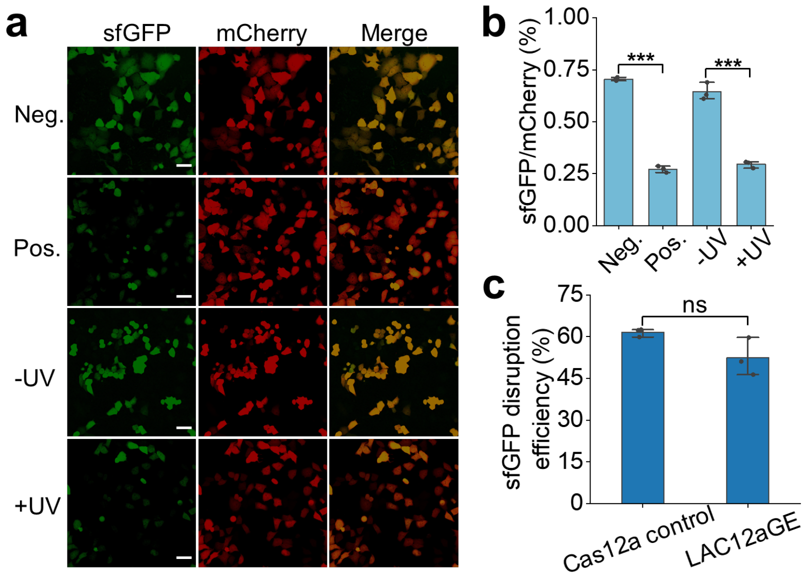

Figure S8. *sfGFP* gene knockout in HeLa cells transfected with LAC12aGE machinery and the fluorescent dual reporter plasmid. (a) Confocal microscopy images corresponding to the fluorescence of sfGFP and mCherry in HeLa cells. Neg., negative control transfected with Cas12a plasmid in the absence of crRNA$_{sfGFP}$. Pos., positive control transfected with Cas12a plasmid and wt-crRNA$_{sfGFP}$. -UV, cells transfected with Cas12a plasmid/OFF-crRNA$_{sfGFP}$ in the absence of UV light. +UV, cells transfected with Cas12a plasmid/OFF-crRNA$_{sfGFP}$ in the presence of UV light. Scale Bar, 50 μm. (b) The relative ratios of sfGFP to mCherry derived from the integrated fluorescence intensities of sfGFP and mCherry displayed in (a). (c) Disruption efficiencies of sfGFP by the Cas12a control and the LAC12aGE machinery, where the sfGFP disruption efficiency (%) by the Cas12a is defined as ($Flu_{Neg.}$ - $Flu_{Pos.}$)/$Flu_{Neg.}$ × 100%, and the disruption efficiency (%) by the LAC12aGE is defined as (Flu_{-UV} - Flu_{+UV})/Flu_{-UV} × 100%. ns, no significant difference; ***, $P < 0.001$. Data represent mean ± SD ($n = 3$).

LAC12aGE machinery-mediated gene cleavage and knockout of the *sfGFP* gene was tested in HeLa cells. Figure S8a shows that sfGFP fluoresence was depleted in both the positive control and the +UV group where cells transfected with Cas12a plasmid/OFF-crRNA$_{sfGFP}$ in the presence of UV light, indicating the Cas12a/crRNA$_{sfGFP}$-stimulated disruption of the *sfGFP* gene. In contrast, mCherry fluorescence remained detectable across all four groups. Figure S8b presents the sfGFP/mCherry fluorescence ratio, which significantly decreases in the postive control group and the +UV group, demonstrating effective and selective disruption of the *sfGFP* gene. The LAC12aGE machinery achieves a 52.4% gene disruption efficiency for *sfGFP* gene in HeLa cells, comparable to the 61.5% efficiency observed in the positive control.

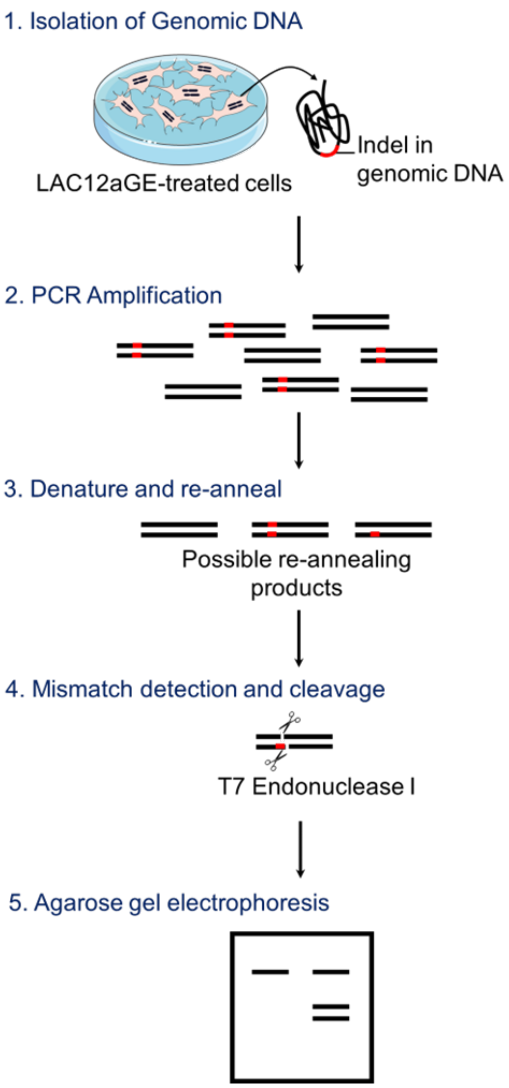

Figure S9. Workflow for detecting indel frequency by T7E1 assay.

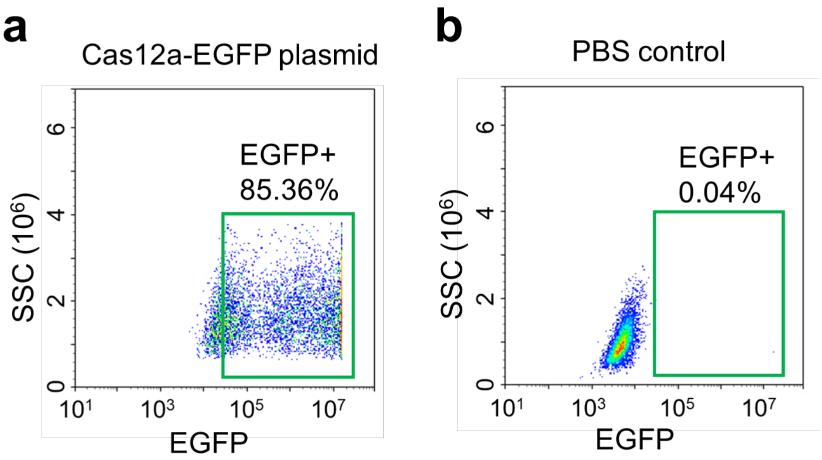

Figure S10. Flow cytometry analysis of transfection efficiency in HEK293T cells following 72 hours of incubation with (a) a Cas12a-EGFP fusion plasmid or (b) PBS (control), delivered using Lipofectamine 3000. FITC fluorescence corresponds to EGFP expression detected in the 488 nm channel, while side scatter (SSC) reflects cellular granularity.

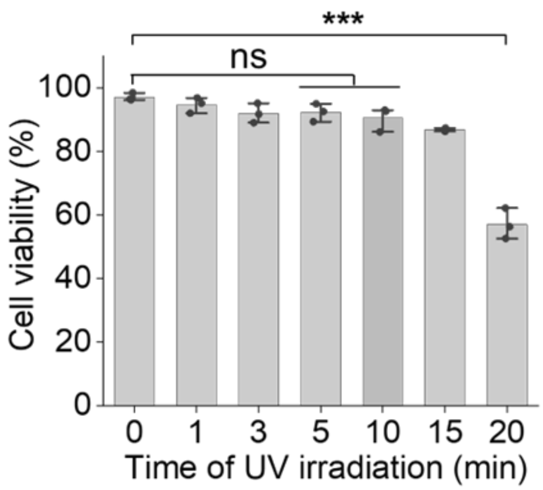

Figure S11. Effects of different time intervals of UV irradiation on the cell viability of HEK293T cells transfected with LAC12aGE machinery. ns, no significant difference; ***, $P < 0.001$. Data represent mean ± SD ($n = 3$).

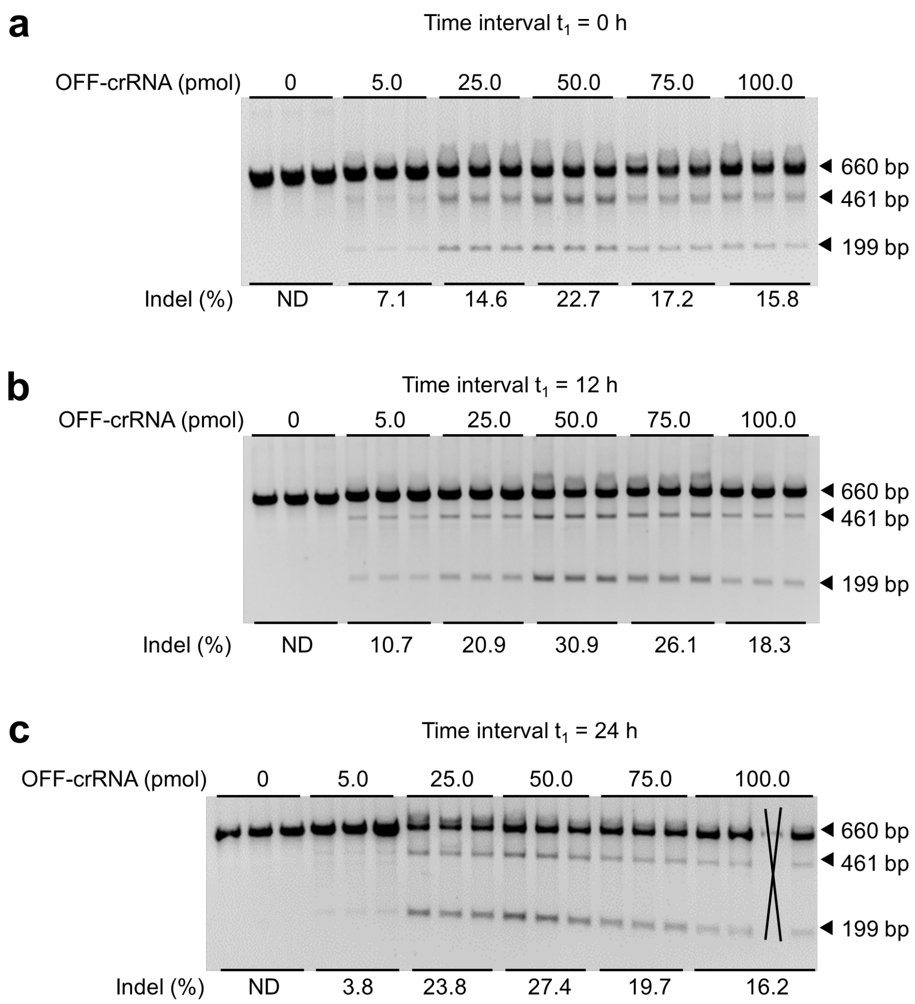

Figure S12. Full-length original gel electrophoresis images corresponding to indel frequencies (%) in Figure 3b resulting from LAC12aGE-mediated gene editing and disruption of *DNMT1* gene in HEK293T cells under variable time intervals of t_1 and OFF-crRNA concentrations: (a) t_1 = 0 h, (b) t_1 = 12 h, and (c) t_1 = 24 h. The intact *DNMT1* gene is 660 bp, which can be cleaved into two fragments composed of 461 bp and 199 bp. Indel values are presented as means. ND indicates not detectable. Unrelated lane is marked with a cross.

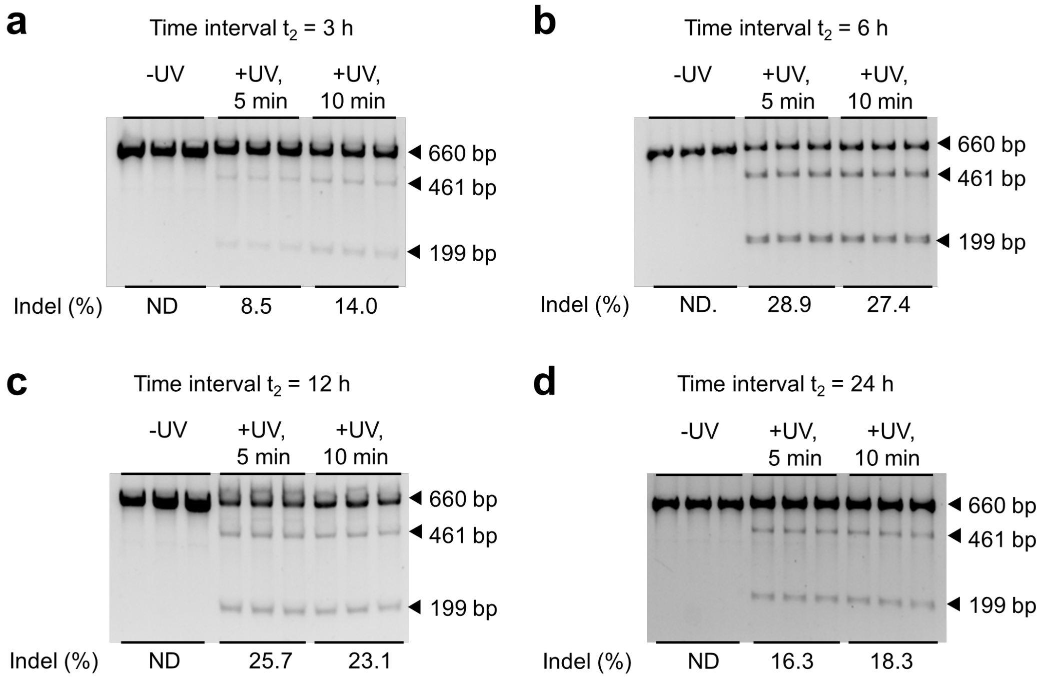

Figure S13. Full-length original gel electrophoresis images corresponding to indel frequencies (%) in Figure 3c resulting from LAC12aGE-mediated gene editing and disruption of *DNMT1* gene in HEK293T cells under variable time intervals of t_2 and UV illumination: (a) t_2 = 0 h, (b) t_2 = 6 h, (c) t_2 = 12 h, and (d) t_2 = 24 h. The intact *DNMT1* gene is 660 bp, which can be cleaved into two fragments composed of 461 bp and 199 bp. Indel values are presented as means. ND indicates not detectable.

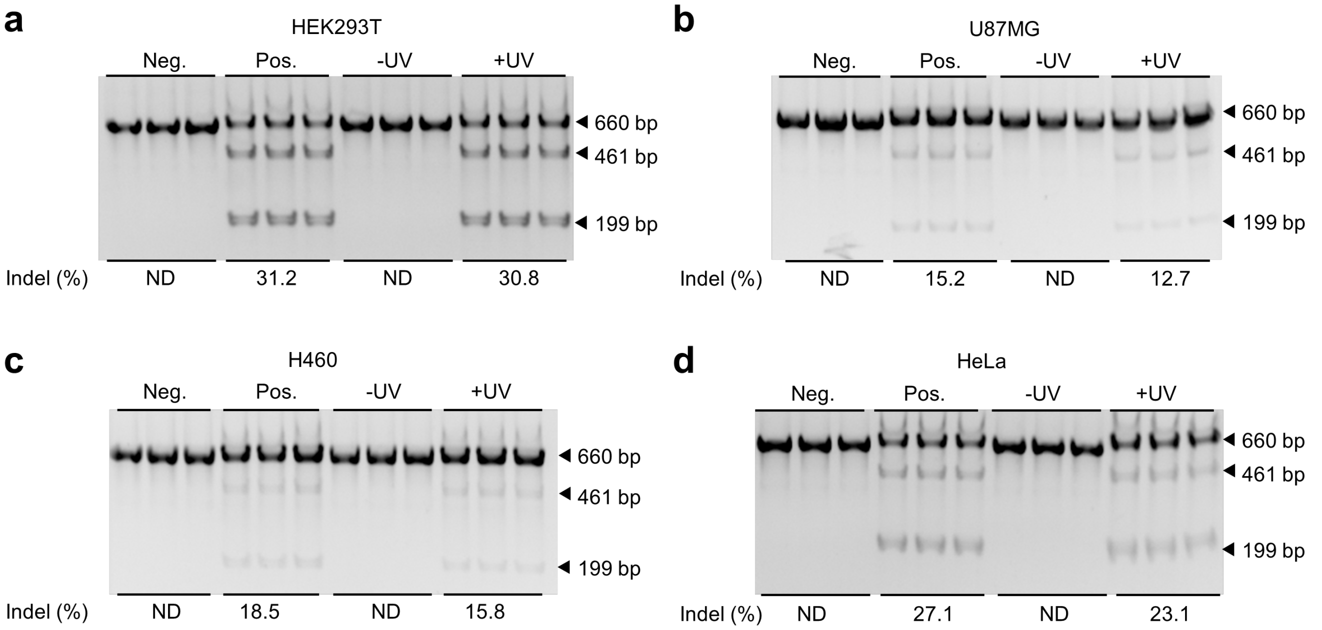

Figure S14. Full-length original gel electrophoresis images corresponding to indel frequencies (%) in Figure 3d resulting from LAC12aGE-mediated gene editing and disruption of *DNMT1* gene in different cell lines: (a) HEK293T, (b) U87MG, (c) H460, and (d) HeLa cells. The intact *DNMT1* gene is 660 bp, which can be cleaved into two fragments composed of 461 bp and 199 bp. Indel values are presented as means. ND indicates not detectable.

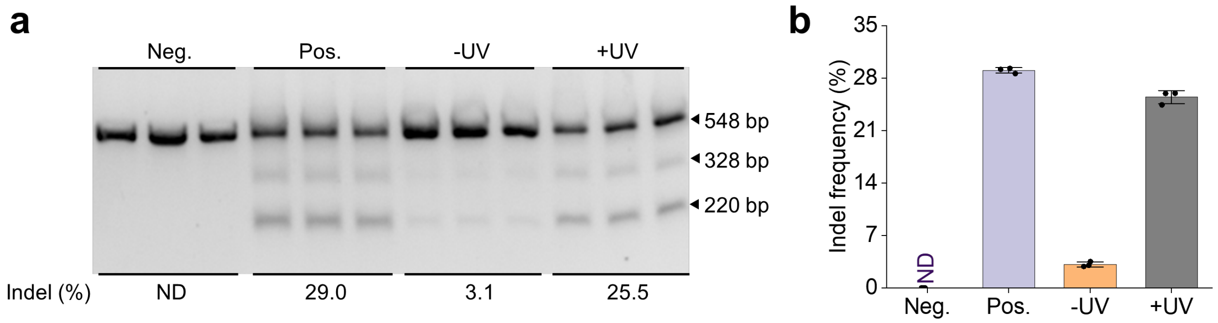

Figure S15. Indel frequencies of LAC12aGE-mediated genome editing activities toward the *VEGFA* gene in HEK293T cells. (a) Full-length original gel electrophoresis images. The intact *VEGFA* gene is 548 bp, which can be cleaved into two fragments composed of 328 bp and 220 bp. (b) Indel frequencies (%) under different treatments. Neg., negative control transfected with Cas12a plasmid in the absence of crRNA$_{VEGFA}$. Pos., positive control transfected with Cas12a plasmid and wt-crRNA$_{VEGFA}$. -UV, cells transfected with Cas12a plasmid/OFF-crRNA$_{VEGFA}$ in the absence of UV light. +UV, cells transfected with Cas12a plasmid/OFF-crRNA$_{VEGFA}$ in the presence of UV light. Data represent mean ± SD (n = 3).

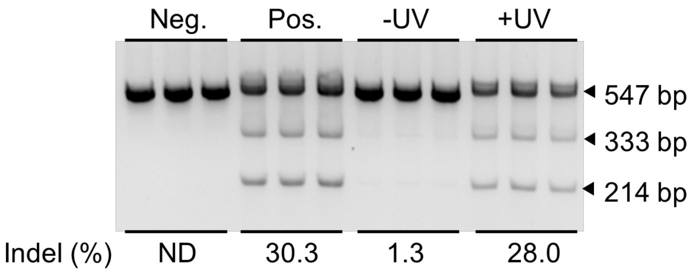

Figure S16. Full-length original gel electrophoresis images corresponding to indel frequencies (%) in Figure 4b resulting from LAC12aGE-mediated genome editing targeting *HGF* gene in HepG2 cells. Neg., negative control transfected with Cas12a plasmid in the absence of crRNA$_{HGF}$; Pos., positive control transfected with Cas12a plasmid and wt-crRNA$_{HGF}$; -UV, cells transfected with Cas12a plasmid/OFF-crRNA$_{HGF}$ in the absence of UV light; +UV, cells transfected with Cas12a plasmid/OFF-crRNA$_{HGF}$ in the presence of UV light. The intact *HGF* gene is 547 bp, which can be cleaved into two fragments composed of 333 bp and 214 bp. Indel values are presented as means. ND indicates not detectable.

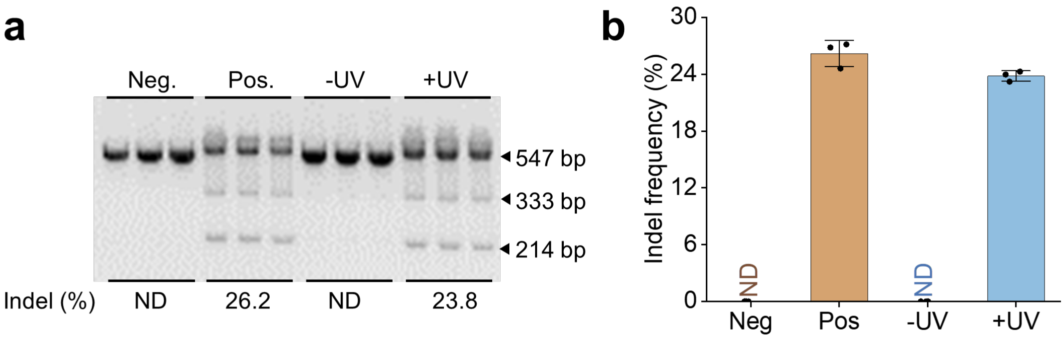

Figure S17. LAC12aGE-mediated genome editing targeting *HGF* gene in Hep3B cells in the absence and presence of UV illumination. (a) Full-length original gel electrophoresis images. The intact *HGF* gene is 547 bp, which can be cleaved into two fragments composed of 333 bp and 214 bp. (b) Indel frequencies (%) under different treatments. Neg., negative control transfected with Cas12a plasmid in the absence of crRNA$_{HGF}$. Pos., positive control transfected with Cas12a plasmid and wt-crRNA$_{HGF}$. -UV, cells transfected with Cas12a plasmid/OFF-crRNA$_{HGF}$ in the absence of UV light. +UV, cells transfected with Cas12a plasmid/OFF-crRNA$_{HFG}$ in the presence of UV light. Data represent means ± SD (n = 3).

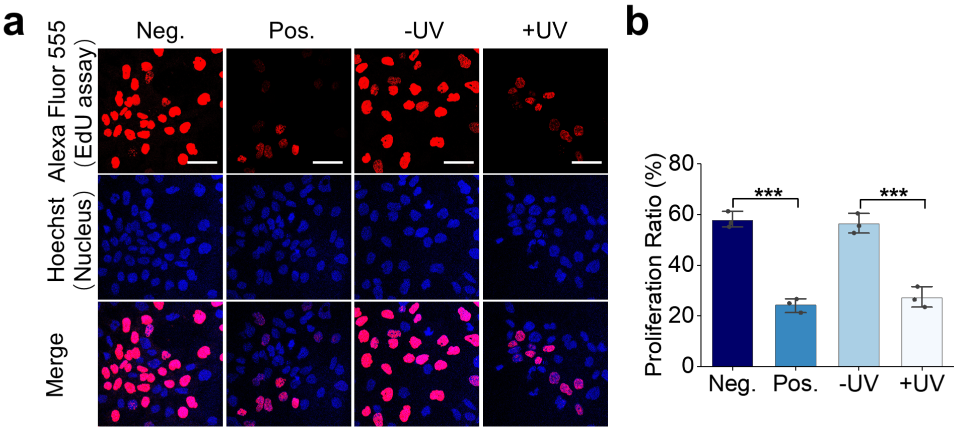

Figure S18. Cell proliferation ability of Hep3B cells in different groups measured by EdU assay after operation of gene editing for 72 hours. (a) Confocal microscopy images of Hep3B cells stained with Alexa Fluor 555 (Edu assay) and Hoechst (nucleus) dyes. Scale Bar, 50 μm. (b) Proliferation ratios of Hep3B cells corresponding to the experiments shown in (a). The cell proliferation ratio (%) is defined as m/n × 100%, where m is the number of Alexa Fluor 555 red fluorescent cells, and n is the total number of Hoechst blue fluorescent cells. Neg., negative control, transfected with Cas12a plasmid in the absence of crRNA$_{HGF}$. Pos., positive control, transfected with Cas12a plasmid and wt-crRNA$_{HGF}$. -UV, cells transfected with Cas12a plasmid/OFF-crRNA$_{HGF}$ in the absence of UV light. +UV, cells transfected with Cas12a plasmid/OFF-crRNA$_{HGF}$ in the presence of UV light. ***, $P < 0.001$. Data represent means ± SD (n = 3).

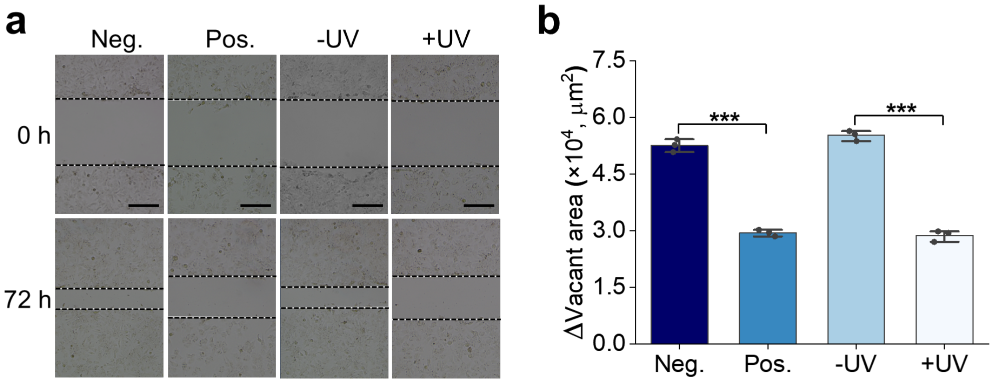

Figure S19. Wound healing assay showing the migration capacities of Hep3B cells in different groups. (a) Microscopy images showing the cell migration capacities of Hep3B cells. Scale Bar, 100 μm. (b) Changes in vacant area separating cells in (a). Neg., negative control, transfected with Cas12a plasmid in the absence of crRNA$_{HGF}$. Pos., positive control, transfected with Cas12a plasmid and wt-crRNA$_{HGF}$. -UV, cells transfected with Cas12a plasmid/OFF-crRNA$_{HGF}$ in the absence of UV light. +UV, cells transfected with Cas12a plasmid/OFF-crRNA$_{HGF}$ in the presence of UV light. ***, $P < 0.001$. Data represent means ± SD ($n = 3$).

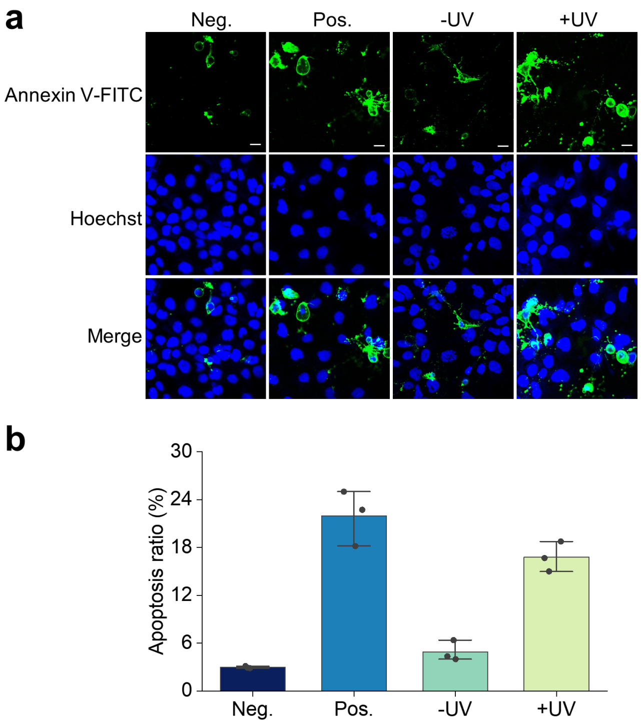

Figure S20. Apoptosis analysis of Hep3B cells in different groups using Annexin V-FITC staining assay. (a) Confocal microscopy images of Hep3B cell stained with Annexin V-FITC (apoptosis) and Hoechst (nucleus) dyes. Scale Bar, 20 μm. (b) Cell apoptosis ratios of Hep3B cells corresponding to the experiments shown in (a). The cell apoptosis ratio (%) is defined as x/y × 100%, where x is the number of Annexin V green fluorescent apoptotic cells, and y is the total number of Hoechst blue fluorescent cells. Neg., negative control, transfected with Cas12a plasmid in the absence of crRNA$_{HGF}$. Pos., positive control, transfected with Cas12a plasmid and wt-crRNA$_{HGF}$. -UV, cells transfected with Cas12a plasmid/OFF-crRNA$_{HGF}$ in the absence of UV light. +UV, cells transfected with Cas12a plasmid/OFF-crRNA$_{HGF}$ in the presence of UV light. Data represent means ± SD (*n* = 3).

Flow cytometry analysis of apoptosis induced by *HGF* gene knockout via the LAC12aGE machinery

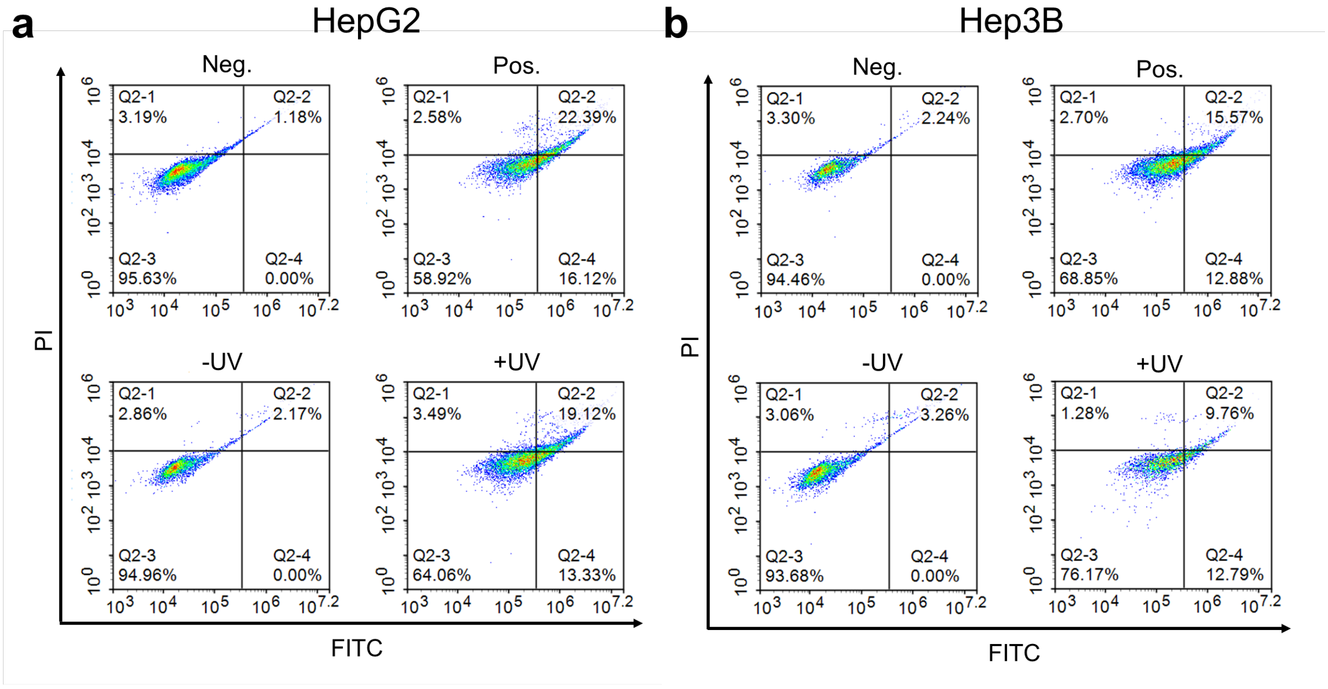

Figure S21. Flow cytometry analysis of apoptosis induced by *HGF* gene knockout in (a) HepG2 and (b) Hep3B cells (1×10^6 cells). Annexin V-FITC was used to stain apoptotic cells, while PI was used to stain dead cells. Neg., negative control, transfected with Cas12a plasmid in the absence of crRNA$_{HGF}$. Pos., positive control, transfected with Cas12a plasmid and wt-crRNA$_{HGF}$. -UV, cells transfected with Cas12a plasmid/OFF-crRNA$_{HGF}$ in the absence of UV light. +UV, cells transfected with Cas12a plasmid/OFF-crRNA$_{HGF}$ in the presence of UV light.

Cell apoptosis resulting from *HGF* gene editing and knockout by the LAC12aGE machinery was further validated through flow cytometry experiments. HepG2 and Hep3B cells (1×10^6) were stained with Annexin V-FITC to probe apoptosis and PI to identify cell death. Cells were treated with the Cas12a/OFF-crRNA$_{HGF}$ machinery either under UV light activation or in the dark, and results were compared to a positive control employing the Cas12a/wt-crRNA$_{HGF}$ machinery. As shown in Figure S21a, light-triggered activation of the Cas12a/OFF-crRNA$_{HGF}$ knockout of *HGF* gene resulted in ca. 32.4% apoptosis of HepG2 cells, which was comparable to the apoptosis induced by the Cas12a/wt-crRNA$_{HGF}$ machinery (38.5%). In Figure S21b, light-triggered activation of the Cas12a/OFF-crRNA$_{HGF}$ knockout of *HGF* gene led to ca. 22.6% apoptosis of Hep3B cells, a level comparable to that induced by the Cas12a/wt-crRNA$_{HGF}$ machinery (28.4%).

RT-qPCR analysis supporting the LAC12aGE-induced *HGF* gene editing in HepG2 and Hep3B cells

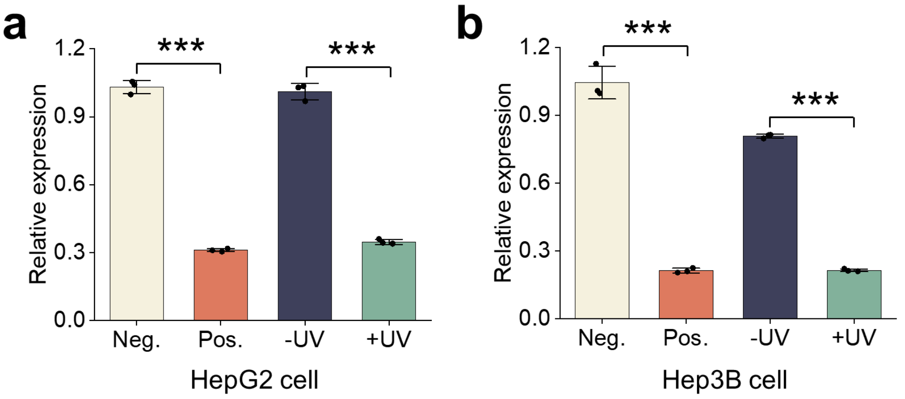

Figure S22. The relative expression levels of *HGF* gene after LAC12aGE-mediated genome editing targeting *HGF* gene in (a) HepG2 cells and (b) Hep3B cells evaluated by RT-qPCR (normalized to negative control). Neg., negative control, transfected with Cas12a plasmid in the absence of $crRNA_{HGF}$. Pos., positive control, transfected with Cas12a plasmid and wt-$crRNA_{HGF}$. -UV, cells transfected with Cas12a plasmid/OFF-$crRNA_{HGF}$ in the absence of UV light. +UV, cells transfected with Cas12a plasmid/OFF-$crRNA_{HGF}$ in the presence of UV light. Data represent means ± SD (n = 3 independent experiments). ***, $P < 0.001$.

Figure S22 presents the *HGF* gene expression levels in HepG2 cells (Figure S22a) and Hep3B cells (Figure S22b) following the LAC12aGE machinery treatment in the absence or presence of light, compared to negative and positive control systems (without or with wt-crRNA), as assessed by RT-qPCR analysis. Evidently, *HGF* gene expression in HepG2 cells decreased by ca. 65.6%, while expression in Hep3B cell was reduced by 73.5%. These results are consistent with the control systems and the indel frequency analysis obtained from T7E1 assay, as displayed in Figure 4b and Figure S17.

Probing the permeability of UV light through mouse skin

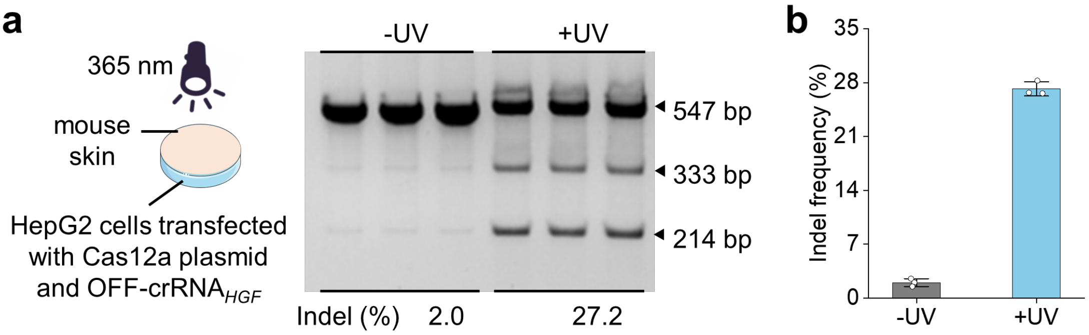

Figure S23. Evaluation of the impact of mouse skin tissue on light-induced genome editing efficiency at the cellular level. (a) Left: Schematic illustration showing UV illumination applied to HepG2 cells transfected with Cas12a plasmid and OFF-crRNA$_{HGF}$, with the light source covered by a layer of mouse skin. Right: Gel electrophoresis analysis of LAC12aGE-mediated genome editing targeting the *HGF* gene in HepG2 cells, with or without UV exposure. The unedited *HGF* gene yields a 547 bp band, whereas successful cleavage generates two fragments of 333 bp and 214 bp. (b) Quantification of indel frequencies (%) in the absence (−UV) or presence (+UV) of illumination following transfection with the Cas12a plasmid and OFF-crRNA$_{HGF}$. Data are presented as mean ± SD (n = 3).

Given the limited permeability of UV light through biological tissues, it was essential to evaluate whether UV irradiation could effectively penetrate mouse skin and activate LAC12aGE-mediated gene editing of the *HGF* gene in HepG2 cells. To this end, a layer of mouse skin tissue was placed over the UV light source, and HepG2 cells transfected with the Cas12a plasmid and OFF-crRNA$_{HGF}$ were exposed to UV illumination (λ = 365 nm, 40 mW/cm^2, 10 min) or kept in the dark as a control. The resulting cell lysates were analyzed via gel electrophoresis (Figure S23). In the UV-irradiated group, efficient gene editing was observed, evidenced by cleavage of the 547 bp *HGF* amplicon into two distinct fragments of 333 bp and 214 bp, corresponding to an indel frequency of 27.2%. In contrast, cells maintained in the absence of UV exposure showed minimal cleavage. These results confirm that UV light can effectively penetrate mouse skin and trigger photodeprotection, thereby enabling gene editing via the LAC12aGE system in underlying cells.

Probing the light-induced deprotection of the OFF-crRNA in the HepG2 tumor-bearing mice

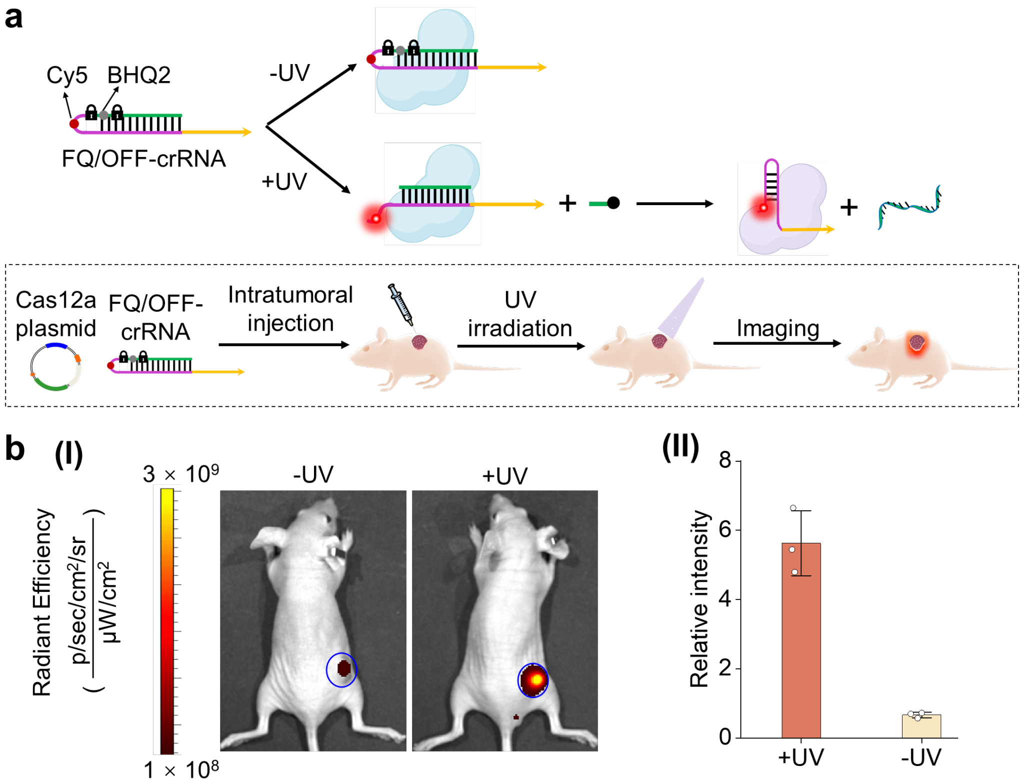

Figure S24. (a) Schematic representation of the Cy5/BHQ2-labeled OFF-crRNA (FQ/OFF-crRNA) and the experimental workflow for evaluating in vivo photodeprotection within mouse tumors. (b) Panel I: Fluorescence imaging of HepG2 tumor-bearing nude mice following intratumoral injection of Cas12a and FQ/OFF-crRNA, with or without UV illumination. Panel II: Relative fluorescence intensities of the tumor sites in (b). Data represent means ± SD (n = 3).

Photochemical uncaging of the *o*-nitrobenzyl phosphate ester-modified OFF-crRNA is a crucial first step in initiating *HGF* gene editing within HepG2 tumors. To verify that uncaging occurs effectively in vivo, the experiment shown in Figure S24a was conducted. In this experiment, a photocaged OFF-crRNA hairpin (FQ/OFF-crRNA) was designed, labeled with a Cy5 fluorophore and a BHQ2 quencher. In its caged configuration, Cy5 fluorescence is quenched by BHQ2. HepG2 tumor-bearing mice were intratumorally injected with the Cas12a plasmid and FQ/OFF-crRNA, followed by UV irradiation (λ = 365 nm, 40 mW/cm^2, 10 min). Successful uncaging is expected to disrupt the hairpin structure, separating Cy5 from BHQ2 and restoring fluorescence. As shown in Figure S24b, panels I and II, strong Cy5 fluorescence was detected in the mouse tumor exposed to UV light, while no signal was observed in the tumor kept in the dark. These results confirm that UV-induced photodeprotection of OFF-crRNA occurs effectively within mouse tumors.

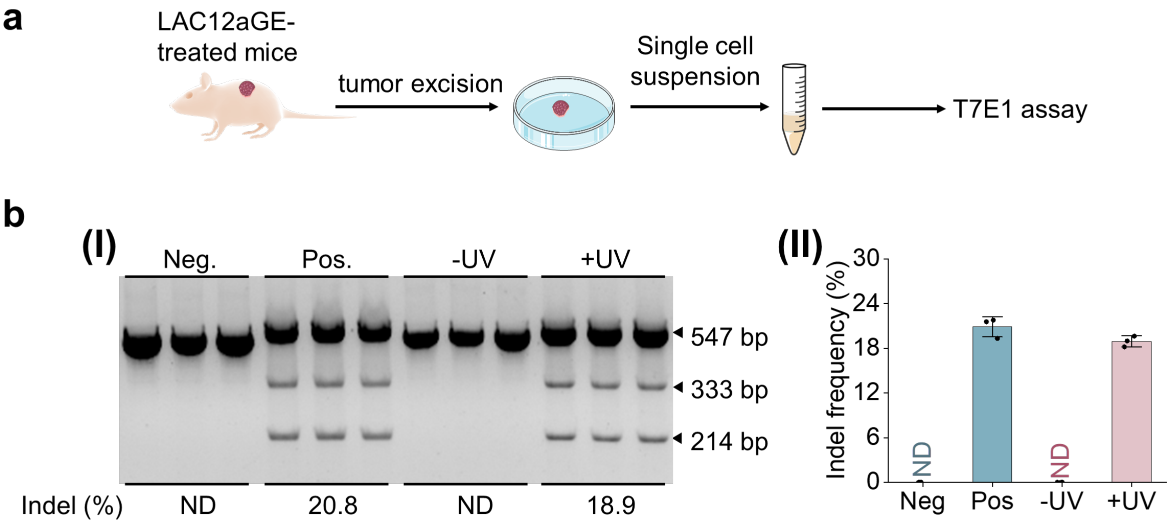

Figure S25. Indel frequencies resulting from LAC12aGE-mediated genome editing of the *HGF* gene in xenograft BALB/c nude mice bearing HepG2 tumors. (a) Schematic illustration of the experimental procedure used to assess indel frequencies at the tissue level. (b) Panel I: Full-length gel electrophoresis images of genomic DNA from tumor tissues. The unedited *HGF* gene yields a 547 bp amplicon, which is cleaved into two fragments of 333 bp and 214 bp upon successful editing. Panel II: Quantification of indel frequencies (%) under various treatment conditions. Neg., negative control treated with Cas12a plasmid in the absence of crRNA$_{HGF}$; Pos., positive control treated with Cas12a plasmid and wt-crRNA$_{HGF}$; -UV, mice treated with Cas12a/OFF-crRNA$_{HGF}$ in the absence of UV light; +UV, mice treated with Cas12a/OFF-crRNA$_{HFG}$ in the presence of UV light. Data represent means ± SD (n = 3).

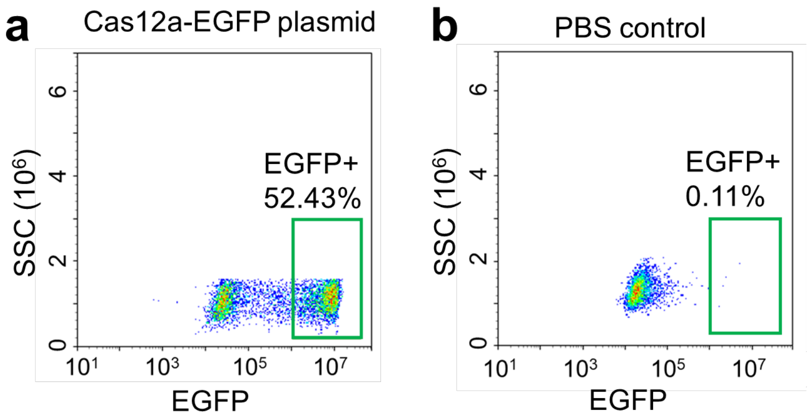

Figure S26. In vivo transfection efficiency assessed by flow cytometry analysis of EGFP-positive cells 72 hours after intratumoral administration in xenograft BALB/c nude mice bearing HepG2 tumors. (a) Cas12a-EGFP fusion plasmid; (b) PBS control. EGFP expression was detected via FITC fluorescence (488 nm channel), while side scatter (SSC) reflects cellular granularity.

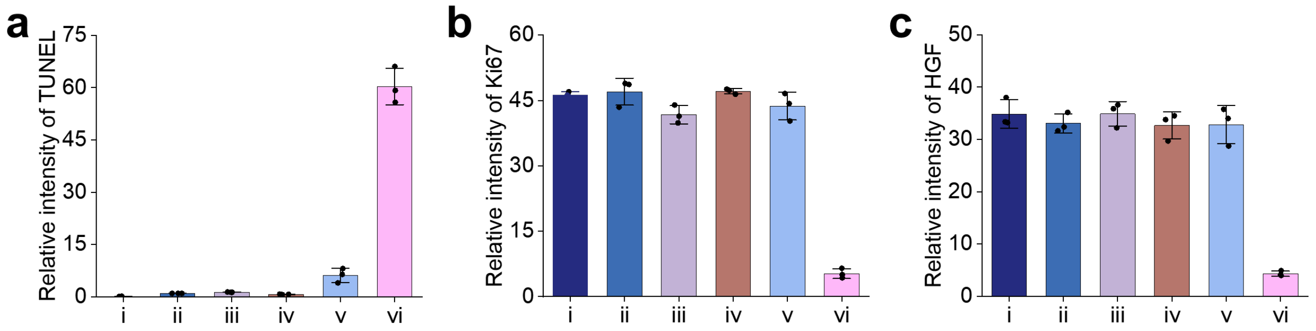

Figure S27. Quantitative fluorescence intensities corresponding to images of tumor tissues from different groups of mice in Figure 6f–h: (a) TUNEL staining, (b) Ki67 staining, and (c) HGF staining. For each group, (i) PBS in the absence of UV light, (ii) PBS in the presence of UV light, (iii) Cas12a/OFF-crRNA$_{Cyan}$ in the absence of UV light, (iv) Cas12a/OFF-crRNA$_{Cyan}$ in the presence of UV light, (v) Cas12a/OFF-crRNA$_{HGF}$ in the absence of UV light, (vi) Cas12a/OFF-crRNA$_{HGF}$ in the presence of UV light. Data represent means ± SD, *n* = 3 independent experiments.

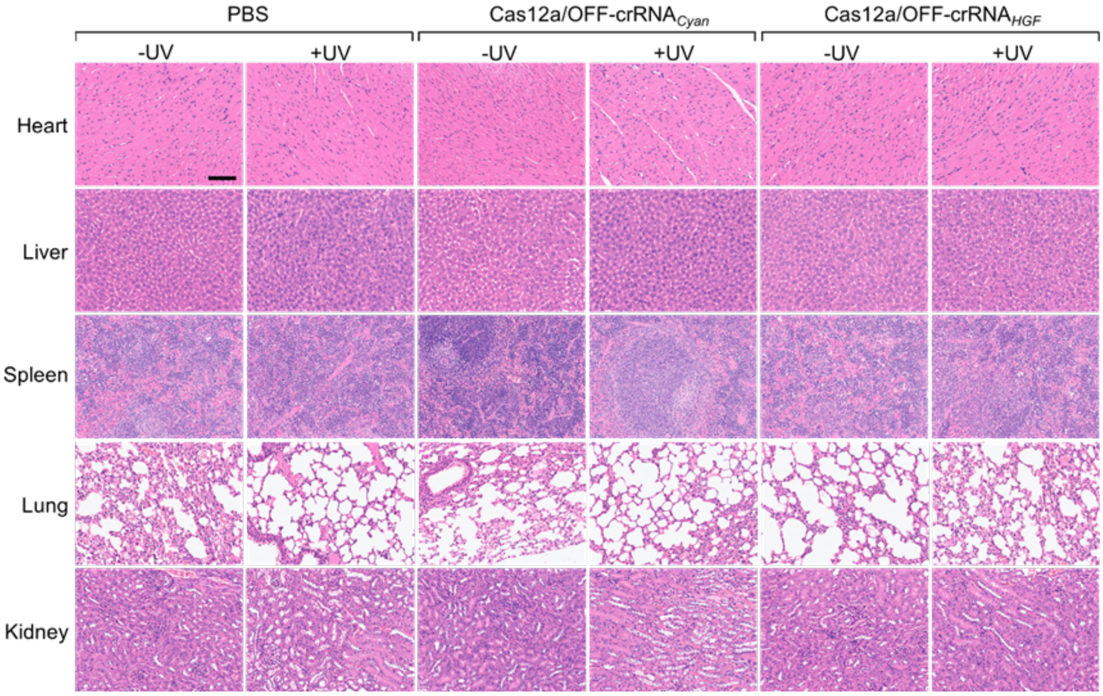

Figure S28. Histology analysis of major organs excised from HepG2 tumor-bearing nude mice after 21-days treatment with PBS, Cas12a/OFF-crRNA$_{Cyan}$, or Cas12a/OFF-crRNA$_{HGF}$, in the absence or presence of UV illumination. Scale bar: 100 µm. Light irradiation: λ = 365 nm, 40 mW/cm^2, 10 min.

References

[1] Y. Zhang, X. Ling, X. Su, S. Zhang, J. Wang, P. Zhang, W. Feng, Y. Y. Zhu, T. Liu, X. Tang, *Angew. Chem. Int. Ed.* **2020**, *59*, 20895–20899; *Angew. Chem.* **2020**, *132*, 21081–21085.
